# Altered Topological Properties of Functional Network Connectivity in Schizophrenia during Resting State: A Small-World Brain Network Study

**DOI:** 10.1371/journal.pone.0025423

**Published:** 2011-09-28

**Authors:** Qingbao Yu, Jing Sui, Srinivas Rachakonda, Hao He, William Gruner, Godfrey Pearlson, Kent A. Kiehl, Vince D. Calhoun

**Affiliations:** 1 The Mind Research Network, Albuquerque, New Mexico, United States of America; 2 Department of Electrical and Computer Engineering, University of New Mexico, Albuquerque, New Mexico, United States of America; 3 Olin Neuropsychiatry Research Center, Hartford, Connecticut, United States of America; 4 Department of Psychiatry, Yale University, New Haven, Connecticut, United States of America; 5 Department of Psychology, University of New Mexico, Albuquerque, New Mexico, United States of America; Beijing Normal University, China

## Abstract

Aberrant topological properties of small-world human brain networks in patients with schizophrenia (SZ) have been documented in previous neuroimaging studies. Aberrant functional network connectivity (FNC, temporal relationships among independent component time courses) has also been found in SZ by a previous resting state functional magnetic resonance imaging (fMRI) study. However, no study has yet determined if topological properties of FNC are also altered in SZ. In this study, small-world network metrics of FNC during the resting state were examined in both healthy controls (HCs) and SZ subjects. FMRI data were obtained from 19 HCs and 19 SZ. Brain images were decomposed into independent components (ICs) by group independent component analysis (ICA). FNC maps were constructed via a partial correlation analysis of ICA time courses. A set of undirected graphs were built by thresholding the FNC maps and the small-world network metrics of these maps were evaluated. Our results demonstrated significantly altered topological properties of FNC in SZ relative to controls. In addition, topological measures of many ICs involving frontal, parietal, occipital and cerebellar areas were altered in SZ relative to controls. Specifically, topological measures of whole network and specific components in SZ were correlated with scores on the negative symptom scale of the Positive and Negative Symptom Scale (PANSS). These findings suggest that aberrant architecture of small-world brain topology in SZ consists of ICA temporally coherent brain networks.

## Introduction

Cognitive dysfunction has been viewed as the core of schizophrenia, a chronic psychotic disorder [1]–[5]. Functional magnetic resonance imaging (fMRI) studies revealed abnormal brain activity in patients with schizophrenia (SZ) during cognitive tasks involving language, memory and attention [6]–[11]. Functional brain disconnectivity has also been considered a hallmark of SZ [12]–[15]. Recently, exploring brain activity in the absence of explicit cognitive or emotional tasks has been a focus of fMRI research. Aberrant resting state networks have become one of the most robust schizophrenia biomarkers which is often revealed by independent components analysis (ICA)[16]–[23].

ICA, which was developed to solve problems similar to the “cocktail party” scenario in which individual voices must be resolved from microphone recordings of many people speaking simultaneously [19], [24], has typically been applied to fMRI data by determining a set of maximally spatially independent brain networks each with associated time courses [25]–[27]. This approach is useful to examine brain activity during resting state in both healthy controls (HCs) and various patient groups [28]–[32]. Jafri et al [33] evaluated functional temporal connectivity among ICA component time courses of resting state fMRI data. The interrelationship among multiple brain networks (components) was defined as functional network connectivity (FNC). They examined differences in FNC between HCs and SZ and found greater occurrences of higher correlation among networks in SZ. However the topological properties of these FNC relationships have not yet been studied.

Studies implementing graph theory to neuroimaging data, especially fMRI, are growing rapidly [31], [34]–[38]. Small-world properties are consistently revealed in human brain networks, which may suggest that the brain generates and integrates information with high efficiency [39]–[44]. Several studies document aberrant small-world network metrics in SZ [45]–[47]. For example, Yu et al [48] discovered altered topological measures in auditory oddball task-related small-world brain networks in SZ. Bloch et al [49] evaluated disrupted modularity in SZ. Several other resting state fMRI studies revealed less hierarchy, less small-world properties, less clustering and less efficiency in SZ [34], [50], [51]. However, all these results were obtained from networks constructed based on brain voxels- or regions-of-interest [52]. The topological properties of brain networks consisting of spatial components in SZ versus HC are yet not known.

The aim of the present study was to identify such differences of small-world network measures in FNC between fMRI data acquired in HCs and SZ during the resting state. We hypothesized that SZ would show abnormal topological properties in this kind of small-world brain networks based on previous studies [33], [53]–[55].

## Materials and Methods

### 1. Ethics Statement

This study has been approved by Hartford Hospital and Yale ethics committee. All subjects gave their written informed consent.

### 2. Participants

Subjects consisted of 19 (7 females) HCs (mean age: 33.9±9.1; range: 24–50) and 19 (4 females) SZ (mean age: 36.5±11.1; range: 21–50). Age showed no significant group difference (P = 0.44). But HCs (15.6±2.2; range: 12–20) have more education years than SZ (12.6±2.6; range: 7–18) (P<0.001). Schizophrenia was diagnosed according to DSM-IV TR criteria on the basis of a structured clinical interview [56] administered by a research nurse and by review of the medical records. All patients had chronic schizophrenia (positive and negative syndrome scale (PANSS) [57]: positive score 16±5; negative score 15±5) and all were taking medication (including the atypical antipsychotic medications aripiprazole, clozapine, risperidone, quetiapine and olanzapine, first-generation antipsychotics including fluphenazine, and miscellaneous mood-stabilizing, hypnotic and anti-cholinergic medications including zolpidem, zaleplon, lorazepam, benztropine, divalproex, trazodone, clonazepam). All participants except 1 healthy control and 2 patients were right-handed. Exclusion criteria included auditory or visual impairment, mental retardation (full scale IQ<70), traumatic brain injury with loss of consciousness greater than 15 min, and presence or history of any central nervous system (CNS) neurological illness. Participants were also excluded if they met criteria for alcohol or drug dependence within the past 6 months or showed a positive urine toxicology screen (screening was for cocaine, opioids including methadone, cannabis, amphetamine, barbiturates, PCP, propoxyphene, and benzodiazepines) on the day of scanning. Healthy participants were free of any DSM-IV TR Axis I disorder or psychotropic medication and had no family histories of Axis I disorders.

### 3. Image acquisition

One 5-min resting state run for each subject was acquired at the Olin Neuropsychiatry Research Center at the Institute of Living/Hartford Hospital on a Siemens Allegra 3T dedicated head scanner equipped with 40 mT/m gradients and a standard quadrature head coil. The functional scans were acquired transaxially using gradient-echo echo-planar-imaging with the following parameters: repeat time (TR) 1.50 s, echo time (TE) 27 ms, field of view 24 cm, acquisition matrix 64×64, flip angle 70°, voxel size 3.75×3.75×4 mm^3^, slice thickness 4 mm, gap 1 mm, 29 slices, ascending acquisition. Six “dummy” scans were acquired at the beginning to allow for longitudinal equilibrium, after which the paradigm was automatically triggered to start by the scanner.

### 4. Preprocessing

FMRI Data were preprocessed using the SPM5 (http://www.fil.ion.ucl.ac.uk/spm/software/spm5/) software package. Data were motion corrected using INRIalign—a motion correction algorithm unbiased by local signal changes [58], spatially normalized into the standard Montreal Neurological Institute (MNI) space, and spatially smoothed with a 10×10×10 mm^3^ full width at half-maximum Gaussian kernel. Following spatial normalization, the data (originally acquired at 3.75×3.75×4 mm^3^) were resliced to 3×3×3 mm^3^, resulting in 53×63×46 voxels.

### 5. Group ICA

Group spatial ICA [55] was conducted for all the data using the infomax algorithm [24]. Data of all 38 participants were decomposed into 75 components using the GIFT software (http://icatb.sourceforge.net/). Single subject time courses and spatial maps were then back-reconstructed [59], [60]. We chose the relatively high model order ICA as previous studies demonstrated that such models yield refined components which correspond to known anatomical and functional segmentations [61]–[64]. The Infomax ICA algorithm was repeated 10 times in ICASSO (http://www.cis.hut.fi/projects/ica/icasso) and resulting components were clustered to estimate the reliability of the decomposition. Fifty-seven components that did not contain large edge effects or ventricles by visual inspection were selected for further analysis. Temporal band-pass filtering (0.01<*f*<0.10 Hz) [65], [66] was performed for component time courses of each subject before computing partial correlations.

### 6. Estimation of inter-component partial correlations

Partial correlation are useful as a measure of connectivity between a given pair of brain regions because they attenuate the contribution of other sources of covariance [67], [68]. Given a set of *N* random variables, the partial correlation matrix is symmetric, where each off-diagonal element is the correlation coefficient between a pair of variables after filtering out the contributions of all other variables included in the dataset [50]. In this study, we evaluated temporal connectivity between each pair of ICs using partial correlation of ICA time courses to reduce the effects of the other 55 brain networks [69], and built undirected graphs respectively for each subject.

The first step was to estimate the sample covariance matrix *S* from the data matrix *Y* = (*x_i_*), *i* = 1,…,57, of observations for each individual. Here *x_i_* was the time courses of each *i* ^th^ component. If we introduce *X* = (*x_j_, x_k_*) to denote the observations in the *j* ^th^ and *k* ^th^ components, *Z = Y\X* denotes the other 55 time courses matrices. Each component of *S* contains the sample covariance value between two components (say *j* and *k*). If the covariance matrix of [*X, Z*] was
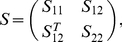
(1)


in which *S_11_* was the covariance matrix of *X*, *S_12_* was the covariance matrix of *X* and *Z* and *S_22_* was the covariance matrix of *Z*, then the partial correlation matrix of *X*, controlling for *Z*, could be defined formally as a normalized version of the covariance matrix [50],
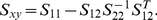
(2)


Finally, a Fisher's *r*-to-*z* transformation [50], [70], [71] was applied to the partial correlation matrix in order to induce normality on the partial correlation coefficients. Connectivity strength [45], [50] of network was computed by absolute z values. Two sample two-tailed t-test was performed to test for group difference in the strength of functional connectivity.

### 7. Graph analysis

An *N*×*N* (*N* = 57 in the present study) binary graph brain network, *G*, consisting of nodes (brain components) and undirected edges (connectivity) between nodes, could be constructed by applying a correlation threshold *T* (Fisher's *r*-to-*z*) to the partial correlation coefficients: 
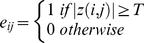
(3)


That is, if the absolute *z*(*i, j*) (Fisher *r*-to-*z* of the partial correlation coefficient) of a pair of components, *i* and *j*, exceeds a given threshold *T*, an edge is said to exist; otherwise it does not exist. We defined the sub-graph *G_i_* as the set of nodes that were the direct neighbors of the *i^th^* node, i.e. directly connected to the *i* ^th^ node with an edge [50]. The degree of each node, *K_i,_ i = 1,2,*…,*57,* was defined as the number of nodes in the sub-graph *G_i_*. The **degree** of connectivity, *K_net_*, of a graph is the average of the degrees of all the nodes in the graph:
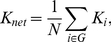
(4)


which is a measure to evaluate the degree of sparsity of a network. The total number of edges in a graph, divided by the maximum possible number of edges *N(N-1)/2*:
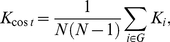
(5)


is called the cost (connection density) of the network.

The **clustering coefficient** of a node was the ratio of the number of existing connections to the number of all possible connections in the subgraph *G_i_* is:
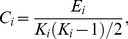
(6)


where *E_i_* is the number of edges in the sub-graph *G_i_* [72], [73]. The clustering coefficient of a network is the average of the clustering coefficients of all nodes:
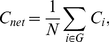
(7)


where *C_net_* is a measure of the extent of the local density or cliquishness of the network.

The **mean shortest path length** of a node was:
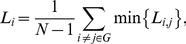
(8)


in which min{*L_i,j_*} is the shortest path length between the *i* ^th^ node and the *j* ^th^ node, and the path length was the number of edges included in the path connecting two nodes. The **characteristic path length** of a network is the average of the shortest path lengths between the nodes:
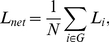
(9)


*L_net_* is a measure of the extent of average connectivity of the network.

*E_global_*, a measure of the **global efficiency** of parallel information transfer in the network, is defined as the inverse of the harmonic mean of the minimum path length between each pair of nodes [43], [74], [75]:
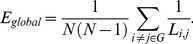
(10)


The **local efficiency** of the *i* ^th^ node can be calculated as follows:
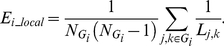
(11)


In fact, since the *i* ^th^ node is not an element of the subgraph *G_i_*, the local efficiency can also be understood as a measure of the fault tolerance of the network, indicating how well each subgraph exchanges information when the index node was eliminated [43]. In addition, based on its definition, it is a measure of the global efficiency of the subgraph *G_i_*. The mean local efficiency of a graph, 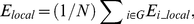
(12)


is the mean of all the local efficiencies of the nodes in the graph.

For more information about the uses and interpretations of the complex brain network measures to see Rubinov and Sporns [35].

### 8. Small-Worldness

Compared with random networks, small-world networks have similar path lengths but higher clustering coefficients, that is *γ = C_net_/C_random_*>1, *λ = L_net_/L_random_*≈1 [73]. These two conditions can also be summarized into a scalar quantitative measurement, small-worldness, *σ = γ/λ*, which is typically >1 for small-world networks [41], [76], [77]. To examine the small-world properties, the values of *C_net_* and *L_net_* of the functional brain network need to be compared with those of random networks. The theoretical values of these two measures for random networks are *C_random_* = *K/N*, and *L_random_*≈ln(*N*)/ln(*K*) [42], [76], [78]. However, as suggested by Stam et al [78], statistical comparisons should generally be performed between networks that have equal (or at least similar) degree sequences; whereas theoretical random networks have Gaussian degree distributions that may differ from the degree distribution of the brain networks that we discovered in this study. To obtain a better control for the functional brain networks, we generated 100 random networks for each degree *K* of each individual network by the same Markov-chain algorithm [44], [79], [80] used in previous studies [50], [81]. And averaged across all 100 generated random networks to obtain a mean *C_random_* and a mean *L_random_* for each cost *K_cost_*.

### 9. Small-World Regime

Since the topological indices of the FNC are computed as a function of threshold, the results can be influenced by differences in the number of edges between the two groups [50], [78]. In this study, we chose thresholds to keep the same number of edges in networks of all participants and investigated the topological properties of the FNC as a function of cost, *K_cost_min_*≤*K_cost_*≤*K_cost_max_*. The range was determined following Liu et al [50] and Liao et al [50], [81]: (1) the minimum cost assures that each network was fully connected with N = 57 nodes. This allowed us to compare the topological properties between the two groups in a way that was relatively independent of the size of the network. (2) the maximum cost was selected to ensure that the brain networks have a lower global efficiency and a larger local efficiency compared to random networks with relatively the same distribution of the degree of connectivity [43]. We selected the small-world regime as 0.351≤*K_cost_*≤0.417 and repeated the full analysis for each value of *K_cost_* with increments of 0.004 (the corresponding number of edges was 560–665 with steps of 7 and the corresponding degree was 19.649–23.333 with steps of 0.246). The range of *K_cost_* was higher than prior studies might indicate standard deviation of partial correlation among brain ICs is higher than among brain regions. The corresponding *z*-score threshold value range for all of the subjects was 0.244–0.513.

### 10. Statistical Analysis

Following Liu et al [50], two sample two-tailed t-tests were performed to test for group differences in *C_net_*, *L_net_*, *E_global_*, *E_local_*, *γ*, *λ* and *σ* at each of the 16 selected *K_cost_* values. A statistical significance level of *P*<0.05 was used. 16×0.05 = 0.80<1 which means there was less than one false-positive result at this [45]. If a significant difference was found, Pearson linear correlation coefficients were used to evaluate the relationship between topological indices and PANSS for each selected cost point in the SZ group. In addition, as each node of the network had its own value of degree (*K_node_*), cluster coefficient (*C_node_*), shortest path length (*L_node_*), global efficiency (*E_node_global_*), and local efficiency (*E_node_local_*), the distribution of components which showed significant differences in these measures were investigated by post-hoc two sample t-tests (*P*<0.05). If significant difference was found for any brain component, Pearson linear correlation coefficients were used to examine the relationship between topological properties of that brain component and PANSS scores in the SZ group.

### 11. Effect of number of components on small-world results

To explore the effect of ICs' number on results of small-world networks, *C_net_*, *L_net_*, *E_local_*, and *E_global_* were computed in HCs and SZ after building networks by changing the number of components as follow. Three more times of group ICA were performed by splitting the fMRI data into 65, 80, 85 ICs. 52, 56, 59 components of interest were selected to build small-world networks respectively. Small-world regimes were 0.324≤*K_cost_*≤0.392 with steps of 0.004; 0.364≤*K_cost_*≤0.432 with steps of 0.004; and 0.317≤*K_cost_*≤0.396 with steps of 0.005. Corresponding number of edges in the networks were 430–520 with steps of 6; 560–665 with steps of 7; and 542–677 with steps of 9.

## Results

### 1. Group ICA and Partial Correlation

Activation of brain regions for each of the selected 57 ICs are shown in supplemental Figures S1, S2, S3, S4, S5, S6, S7, S8, S9, S10, S11, S12, S13, S14, S15, S16, S17, S18, S19, S20, S21, S22, S23, S24, S25, S26, S27, S28, S29, S30, S31, S32, S33, S34, S35, S36, S37, S38, S39, S40, S41, S42, S43, S44, S45, S46, S47, S48, S49, S50, S51, S52, S53, S54, S55, S56, S57. Figure 1 shows the mean connectivity matrix which was calculated by averaging the *N*×*N* (*N* = 57 in this study) absolute partial correlation matrix of all the subjects within each group. Group difference of connectivity strength was not significant which means we did not repeat the finding of Jafri et al [33] which found higher correlations in SZ than controls in some of the component pairs. However, Jafri et al used 7 components of interest from all 30 components, and statistical analysis was performed on a constrained maximal lagged correlation between components. In this study, 57 components were selected from all 75 components. In addition, connectivity was computed by partial correlation. Both of the different methods may play a role to cause the inconsistent results.

**Figure 1:**
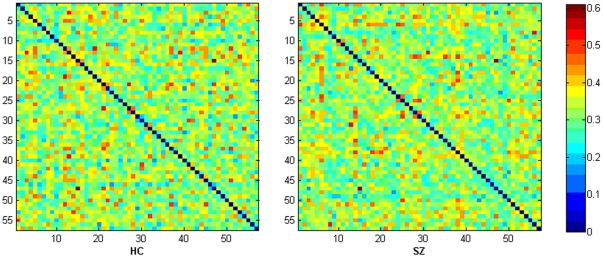
Mean absolute *z*-score matrices for HCs and SZ. Each figure shows a 57×57 square matrix, where each entry indicates the mean strength of the connectivity between each pair of components which were organized in the same sequence as in supplemental material. The diagonal running from the upper left to the lower right is intentionally set to zero.

### 2. Small-world network metrics

**Figure 2:**
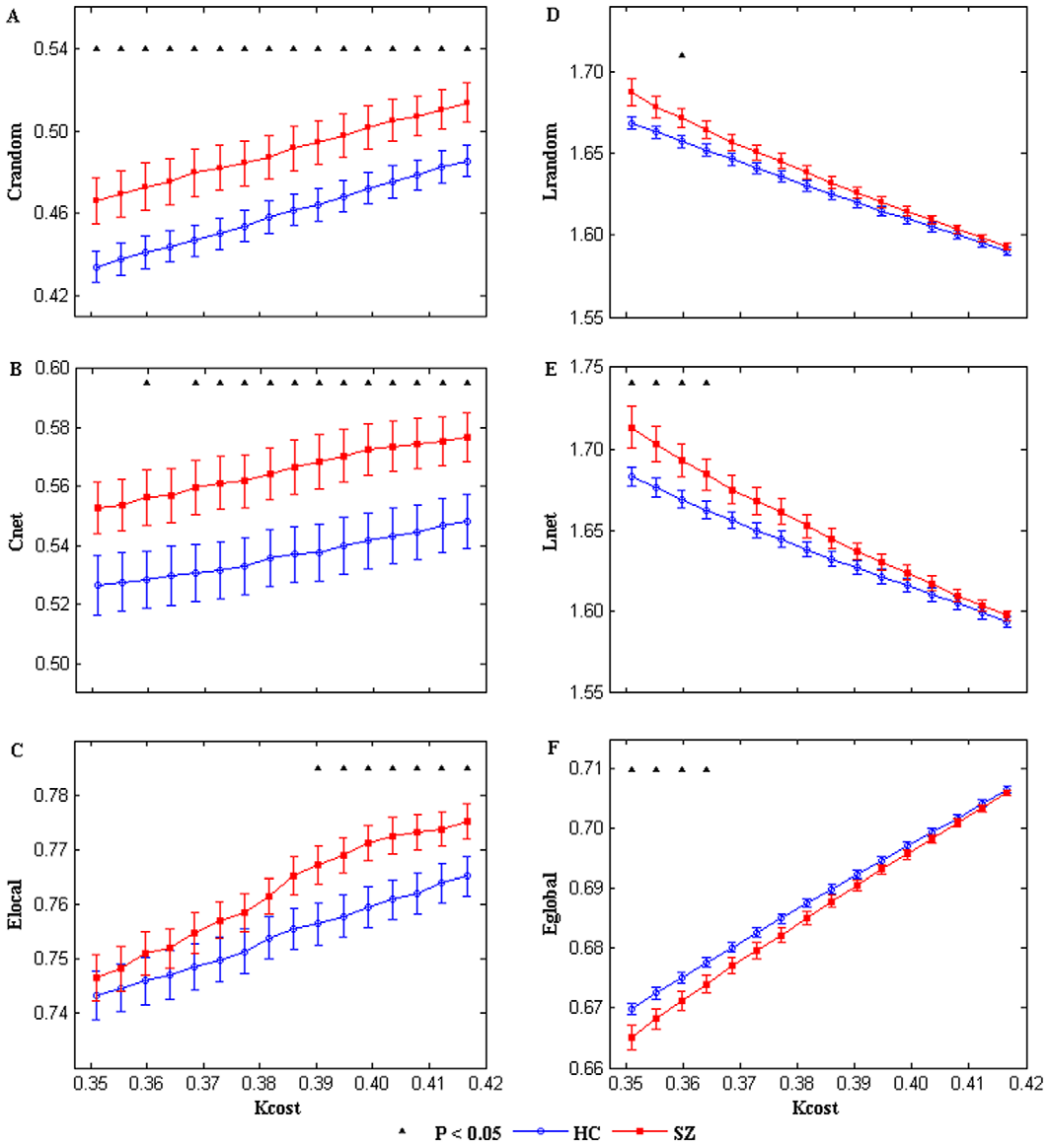
Network metrics of each group. Mean clustering coefficient (B), local efficiency (C), characteristic path length (E) and global efficiency (F) of the FNC for HCs (blue circles) and SZ (red squares) as a function of cost *K_cost_*. For comparison, *C_random_* (A) and *L_random_* (D) are also shown. Error bars correspond to standard error of the mean (across 19 subjects of each group). Black triangles indicate where the group difference is significant (two sample t-test, *P*<0.05, uncorrected).

For topological indices as a function of cost, as cost increases, the clustering coefficient, local efficiency and global efficiency also increase whereas the characteristic path length decreases because more and more edges are added into the network. As shown in Figure 2, two sample t-tests indicated that clustering coefficients were higher (*P*<0.05, uncorrected) in SZ at most of the cost points; local efficiencies were higher in SZ only at higher cost values (7 highest values of the selected cost); whereas the character path length and global efficiency showed significant group difference at lower cost values (4 lowest values of the selected cost), SZ had higher characteristic path length and lower global efficiency. After changing the number of components (52, 56, 59 ICs from total of 65, 80, 85), SZ were showing the same trend for all the network properties. And the group differences of *C_net_* and *E_local_* at some *K_cost_* points were significant (*P*<0.05, uncorrected) or marginally significant (*P*<0.1, uncorrected).

The small-world attribute was evident in the FNC of both groups. *γ* was significantly greater than 1 while *λ* was near to 1 over the whole range of *K_cost_*. Because *C_random_* and *L_random_* show similar group differences as Cnet and Lnet (see Figure 2), no statistical significant differences in the values of *γ*, *λ* or *σ* between the two groups were found when the same cost was used.

### 3. Relationships between network measures and PANSS scores in SZ

**Figure 3:**
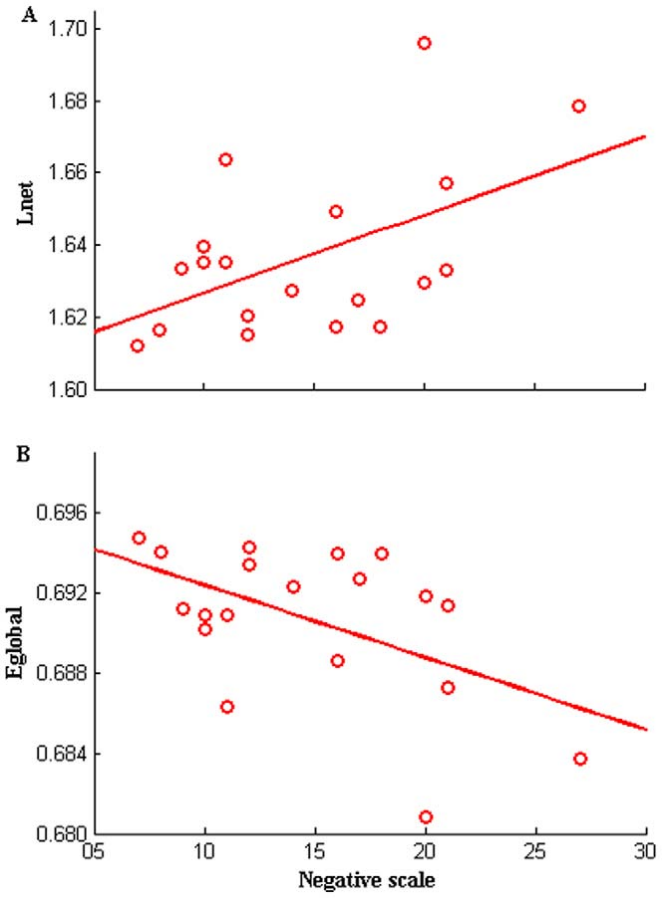
Scatter plots of path length and global efficiency in SZ. Scatter plots with trend line showing characteristic path length (L_net_) (A) and global efficiency (E_global_) (B) of the FNC as a function of PANSS negative scale at a selected cost (*K_cost_* = 0.390) in SZ. Pearson correlation coefficient for L_net_ (*R* = 0.513, *P* = 0.025, uncorrected), E_global_ (*R* = −0.515, *P* = 0.024, uncorrected).

Significant (*P*<0.05, uncorrected) correlations between topological metrics and PANSS values in SZ were only found between characteristic path length (*L_net_*), global efficiency (*E_global_*) and negative scale score by Pearson correlation analysis. *L_net_* was positively correlated with negative scale of PANSS whereas *E_global_* was negatively correlated with the PANSS negative scale. Figure 3 shows the patterns at a typical cost point (*K_cost_* = 0.390).

### 4. Distribution of components in which topological metrics are altered in SZ

**Figure 4:**
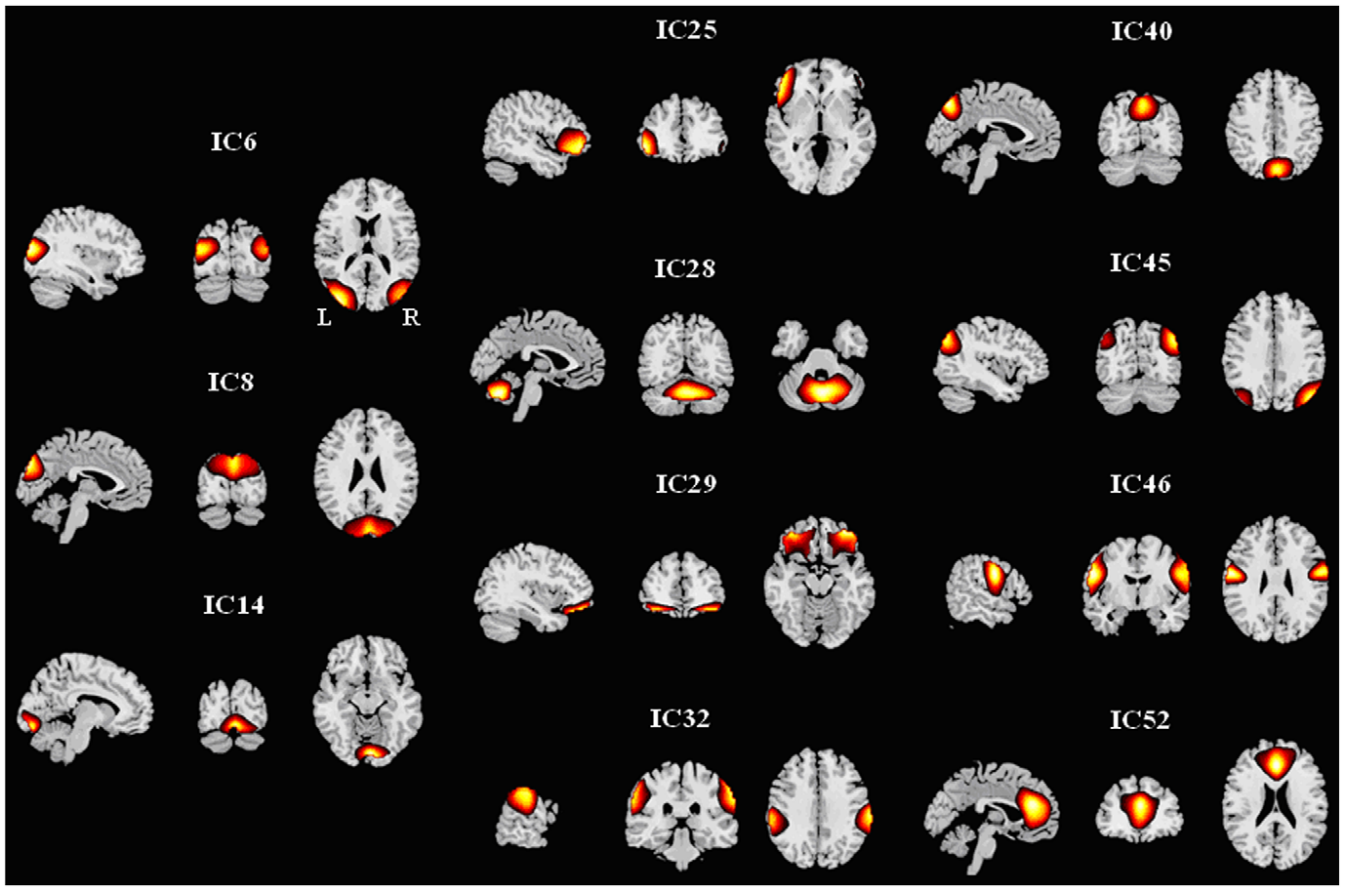
Activation z-maps of the brain components in which graph metrics are altered significantly in SZ (group ICA result).

**Figure 5:**
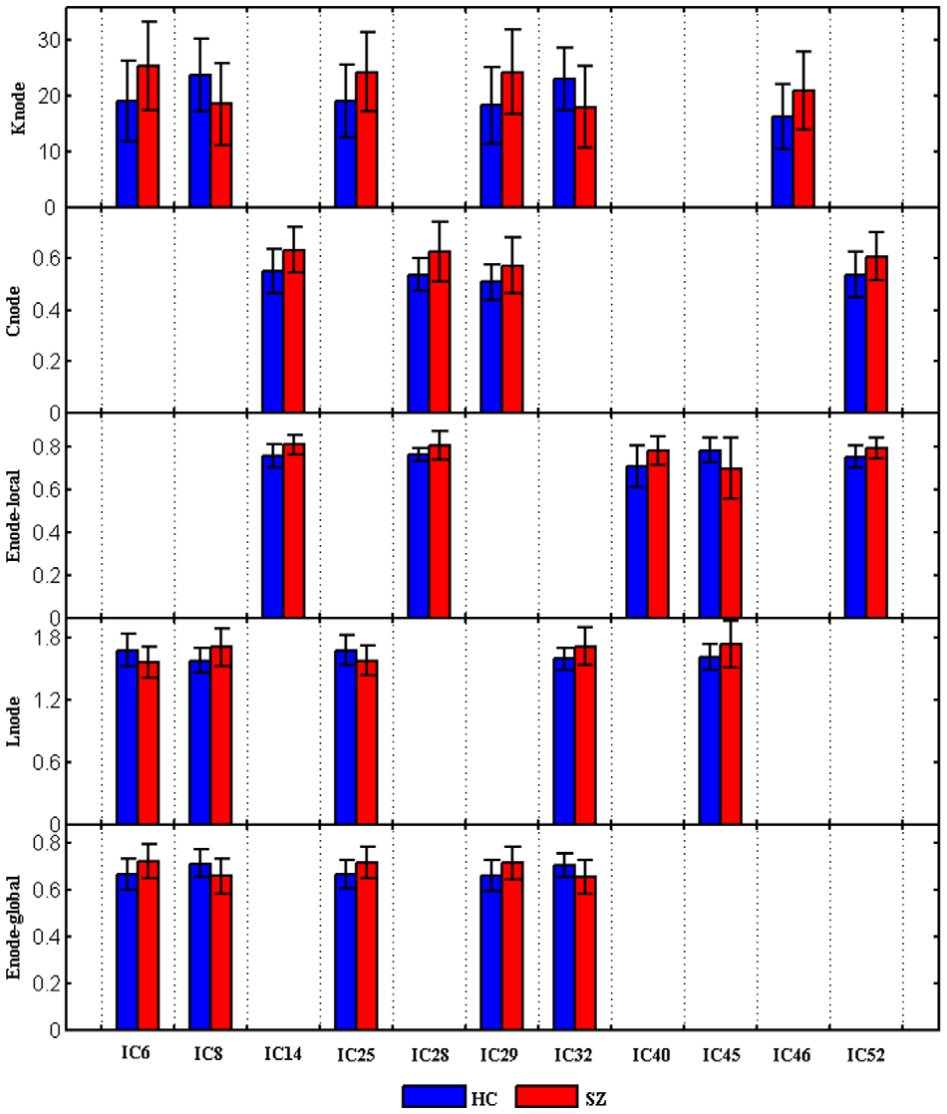
Mean value of topological properties which showed group difference. Values of K_node_, C_node_, E_node_local_, L_node_, E_node_global_ altered significantly (*P*<0.05, uncorrected) in SZ at a selected cost (*K_cost_* = 0.382) are shown. The color of the bar indicates the group and the height of the bar indicates the mean value of the relative measurement for the two groups. Error bars correspond to standard deviation.

**Figure 6:**
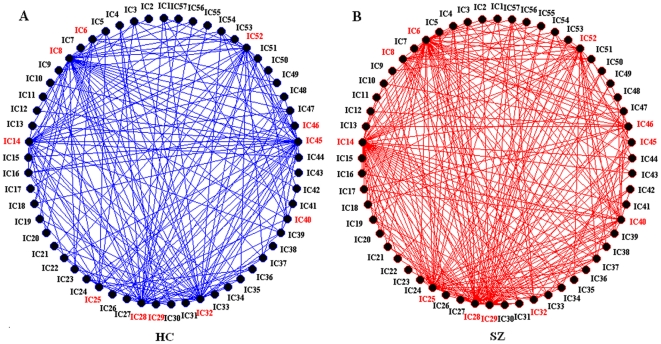
Examples of network connection patterns for HCs and SZ. The networks were built from mean absolute *z*-score (partial correlation) matrices (Figure 1) at a typical cost (*K_cost_* = 0.382, corresponding *z*-score threshold values: HCs 0.318; SZ 0.336). Red named nodes indicates graph indices were altered in SZ for those components. Only edges which were connected to any of those eleven nodes are shown.

When using two sample t-tests to explore statistical differences in the topological properties including degree, clustering coefficient, local efficiency, shortest path length and global efficiency between SZ and HC for each of the 57 components at each selected cost, we found significant differences (*P*<0.05, uncorrected) in eleven components (IC6, IC8, IC14, IC25, IC28, IC29, IC32, IC40, IC45, IC46 and IC52) which involve frontal, parietal, occipital and cerebellar brain areas (Figure 4 shows the activation of these components). Although the results do not pass false discovery rate (FDR) correction (*P*<0.05) for multiple comparisons of 57 nodes, these tests are implemented after finding significant group differences in the metrics of the whole network. Thus the node specific comparisons are useful to report as post-hoc tests. Figure 5 displays the trends of relative parameters for those individual components at a typical cost (*K_cost_* = 0.382). For an example of network connection patterns in HC and SZ see Figure 6 (edges which were connected to any of the eleven nodes are shown).

### 5. Relationships between network properties of individual components and PANSS scores in SZ

**Figure 7:**
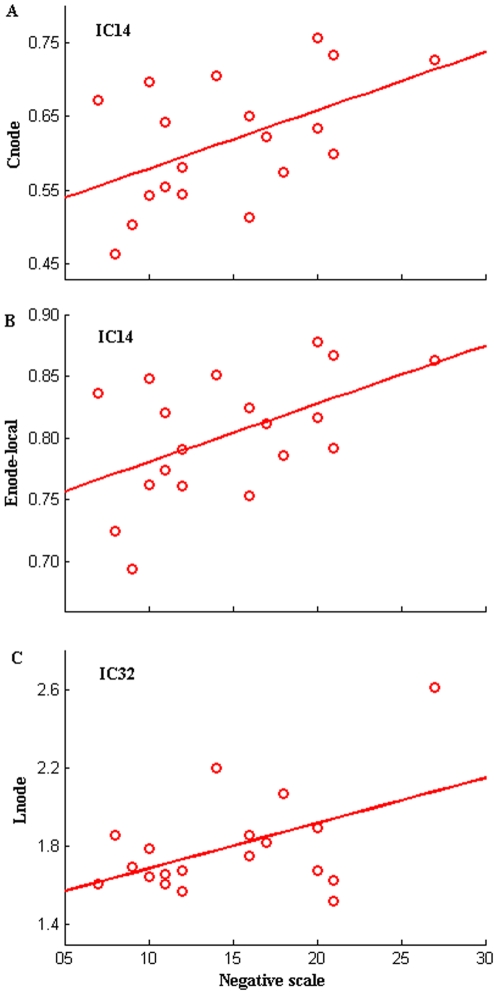
Scatter plots of clustering coefficient and characteristic path length for individual components in SZ. Scatter plots with trend line showing C_node_ of IC14 (A) (*R* = 0.504, *P* = 0.028, uncorrected), E_node_local_ of IC14 (B) (*R* = 0.511, *P* = 0.025, uncorrected) and L_net_ of IC32 (C) (*R* = 0.482, *P* = 0.037, uncorrected) as a function of negative scale of PANSS at a selected cost (*K_cost_* = 0.351) in SZ.

Based on Pearson correlation coefficients, shortest path length and local efficiency of IC14, shortest path length of IC32 were significantly (*P*<0.05, uncorrected) positively correlated with negative scale of PANSS. Patterns at a typical cost point (*K_cost_* = 0.351) were shown in Figure 7.

## Discussion

In the present study, resting state fMRI data for both HC and SZ were decomposed into spatially independent components by group ICA. Undirected graphs were built based on partial correlation matrices which were computed using ICA time courses. The brain networks showed “small-world” patterns in the selected range of cost in both groups that was consistent with previous studies that found small-world features in complex brain networks consisting of brain regions [36], [44]. The finding that topological properties were altered in SZ and that several metrics were significantly correlated with negative PANSS scores in SZ provides further evidence for aberrant FNC associated with this disease [33]. One novel aspect of this study is that all results were obtained from brain topology consisting of ICA temporally coherent brain networks.

### 1. Altered topological metrics in patients

Short characteristic path lengths and high global efficiencies have been demonstrated to promote effective interactions between and across different cortical regions [42], [43], [50]. In our study, characteristic path length showed significant higher value and global efficiency showed lower value only at lower cost points (see Figure 2), possibly indicating that information interactions between temporally interconnected brain components were slower and less efficient in SZ when the whole network of FNC was sparse.

Clustering coefficients were significantly increased at most of the selected cost points and local efficiencies were increased only at the higher cost points in SZ (see Figure 2) in the networks constructed based on brain components in this study. This finding implied relatively dense local connectedness and robust local information processing of the brain networks in SZ [50], [74], [82] suggesting abnormal FNC in schizophrenia. The findings that clustering coefficients and local efficiencies were higher in SZ are consistent with previous studies which involved patients with other brain disorders. For example, Wang et al [83] found increased local efficiency in children with ADHD (attention-deficit/hyperactivity disorder); He et al [84] revealed increased clustering coefficient in Alzheimer's disease. However, as far as we know, all prior studies involving schizophrenia in which networks were constructed based on brain regions found less clustering coefficient and less local efficiency in SZ [34], [45], [50], [51]. It is notable that this is the first work which finds increased clustering coefficient and local efficiency in schizophrenia. Moreover, that HCs and SZ showed similar small-worldness values distinguished our results from prior brain region-based graph studies [45], [50]. Reasons for getting the distinct results are not clear, but the different graph building method (ICs-based graphs) of this study may play a role.

### 2. Distribution of altered brain components

Consistent with previous findings [45], [50], only some nodes were altered in SZ. The network metrics of some components involving frontal, parietal, occipital and cerebellar brain areas were significantly altered in SZ (see Figure 4, 5). For example, the degree of connectivity for IC8 (occipital region) was smaller in SZ indicating a lower connectivity between this component and other brain components. Our findings that SZ show aberrant connectivity in vision (IC6, IC8, IC14), motor (IC28, IC32, IC46), attention (IC25) and default (IC40, IC45, IC52) networks are consistent with previous studies which reported abnormal activation [85], [86] or disturbed connectivity in those brain regions in SZ [33], [87]–[89].

### 3. Relationship between topological measurements and PANSS scores in SZ

Interestingly, we found characteristic path length and global efficiency of the whole brain network were correlated with PANSS negative scale values in SZ. Higher negative scale scores were associated with longer character path length and lower global efficiency (Figure 3). These might indicate the more severe these symptoms, the lower information interactions among brain components. In addition, clustering coefficient, local efficiency of IC14 (occipital region) and shortest path length of IC32 (parietal region) were correlated with negative PANSS scores in SZ (see Figure 7). Higher negative scale scores were associated with a higher clustering coefficient, higher local efficiency of IC14 (occipital region) and longer shortest path length of IC32 (parietal region). These findings are in line with studies which found psychopathology is associated with aberrant intrinsic organization of functional brain networks in schizophrenia [21] and provide further evidence for this illness as a disconnection syndrome [50], [90]–[93].

### 4. Methodological limitations

The main limitation of this study is our use of a statistical significance level of *P*<0.05 (uncorrected). We did not use any stringent type I error control such as Bonferroni or false discovery rate correction especially for the results of nodal level analysis. Further studies could increase the statistical power by increasing the sample of subjects. The second possible confound is medication use in patients. It is not clear if medication in SZ alters graph parameters. In addition, this study examined the measures of not weighted but only binarised networks. Further studies are required to address these considerations.

For nodal definition, compared with previous studies which used a predefined anatomical template, ICA-based nodes are data-driven. Each IC consisted of intrinsic connected brain voxels which share the same time course. By using this kind of graphs, altered topological metrics among intrinsic connected brain networks (but not predefined brain regions which are not intrinsic connected) were revealed. However, there are also some disadvantages by using ICs as graph nodes. For example, the potential for artificial splitting of a network into spurious sub-networks when the ICA dimensionality is highly chosen (here is 75 components based on 5 minutes of resting state data); the potential for lack of coverage of certain cortical regions or over-representation (spatial overlap) of certain other cortical regions; 57 non-artifactual components were selected arbitrarily. The effect of specified number of components on small-world results should be further studied by recruiting larger sample of subjects in future.

### 5. Conclusions

To our knowledge, this is the first study using ICA and graph theory methods to explore abnormal small-world brain network properties in SZ during the resting state. Small-world topological metrics including clustering coefficient, local efficiency, characteristic path length and global efficiency of the graph built based on FNC were altered in SZ. Characteristic path length and global efficiency measures were correlated with negative scale scores on the PANSS. The network parameters of some individual brain components involving frontal, parietal and occipital areas were disturbed in SZ. These findings provide further evidence for aberrant FNC in SZ and expand our understanding of brain disconnection in schizophrenia.

## Supporting Information

Figure S1 Spatial map of IC1.
(TIF)

Figure S2 Spatial map of IC2.
(TIF)

Figure S3 Spatial map of IC3.
(TIF)

Figure S4 Spatial map of IC4.
(TIF)

Figure S5 Spatial map of IC5.
(TIF)

Figure S6 Spatial map of IC6.
(TIF)

Figure S7 Spatial map of IC7.
(TIF)

Figure S8 Spatial map of IC8.
(TIF)

Figure S9 Spatial map of IC9.
(TIF)

Figure S10 Spatial map of IC10
(TIF)

Figure S11 Spatial map of IC11
(TIF)

Figure S12 Spatial map of IC12
(TIF)

Figure S13 Spatial map of IC13
(TIF)

Figure S14 Spatial map of IC14
(TIF)

Figure S15 Spatial map of IC15.
(TIF)

Figure S16 Spatial map of IC16.
(TIF)

Figure S17 Spatial map of IC17.
(TIF)

Figure S18 Spatial map of IC18.
(TIF)

Figure S19 Spatial map of IC19.
(TIF)

Figure S20 Spatial map of IC20.
(TIF)

Figure S21 Spatial map of IC21.
(TIF)

Figure S22 Spatial map of IC22.
(TIF)

Figure S23 Spatial map of IC23.
(TIF)

Figure S24 Spatial map of IC24.
(TIF)

Figure S25 Spatial map of IC25.
(TIF)

Figure S26 Spatial map of IC26.
(TIF)

Figure S27 Spatial map of IC27.
(TIF)

Figure S28 Spatial map of IC28.
(TIF)

Figure S29 Spatial map of IC29.
(TIF)

Figure S30 Spatial map of IC30.
(TIF)

Figure S31 Spatial map of IC31.
(TIF)

Figure S32 Spatial map of IC32.
(TIF)

Figure S33 Spatial map of IC33.
(TIF)

Figure S34 Spatial map of IC34.
(TIF)

Figure S35 Spatial map of IC35.
(TIF)

Figure S36 Spatial map of IC36.
(TIF)

Figure S37 Spatial map of IC37.
(TIF)

Figure S38 Spatial map of IC38.
(TIF)

Figure S39 Spatial map of IC39.
(TIF)

Figure S40 Spatial map of IC40.
(TIF)

Figure S41 Spatial map of IC41.
(TIF)

Figure S42 Spatial map of IC42.
(TIF)

Figure S43 Spatial map of IC43.
(TIF)

Figure S44 Spatial map of IC44.
(TIF)

Figure S45 Spatial map of IC45.
(TIF)

Figure S46 Spatial map of IC46.
(TIF)

Figure S47 Spatial map of IC47.
(TIF)

Figure S48 Spatial map of IC48.
(TIF)

Figure S49 Spatial map of IC49.
(TIF)

Figure S50 Spatial map of IC50.
(TIF)

Figure S51 Spatial map of IC51.
(TIF)

Figure S52 Spatial map of IC52.
(TIF)

Figure S53 Spatial map of IC53.
(TIF)

Figure S54 Spatial map of IC54.
(TIF)

Figure S55 Spatial map of IC55.
(TIF)

Figure S56 Spatial map of IC56.
(TIF)

Figure S57 Spatial map of IC57.
(TIF)
