# Supplementary figures and images for: Altered Topological Properties of Functional Network Connectivity in Schizophrenia during Resting State: A Small-World Brain Network Study
Source: PLoS One. 2011 Sep 28;6(9):e25423. doi: 10.1371/journal.pone.0025423 (PMC3182226; doi:10.1371/journal.pone.0025423)

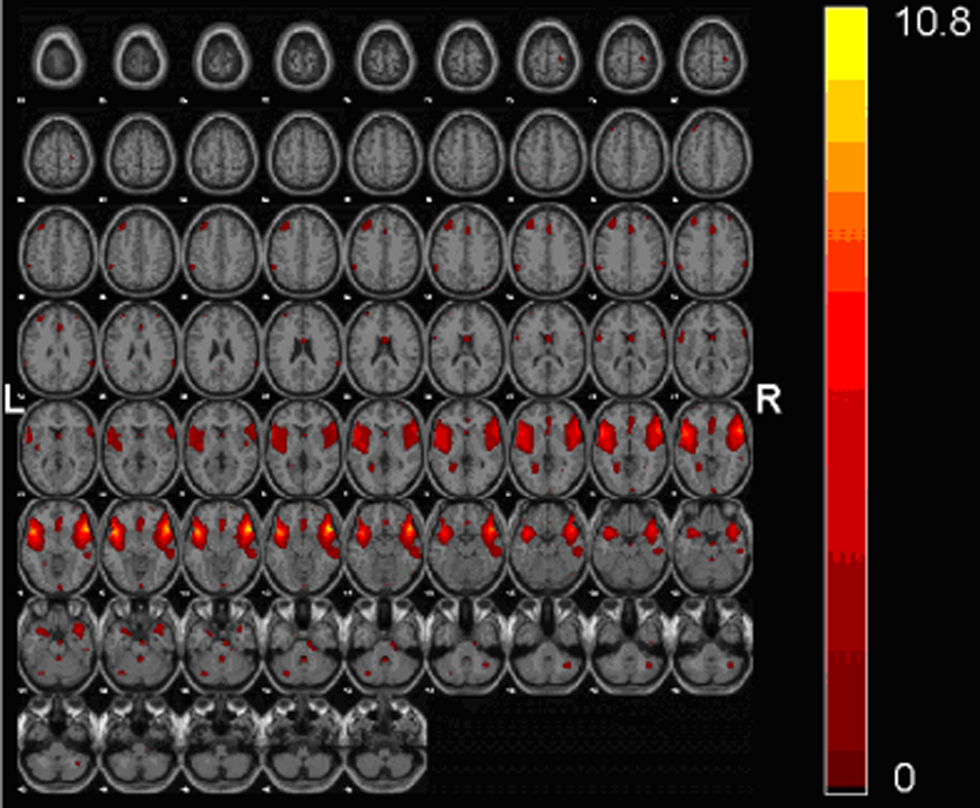

Supplement: Figure S1 — Spatial map of IC1. (TIF) [file pone.0025423.s001.tif]

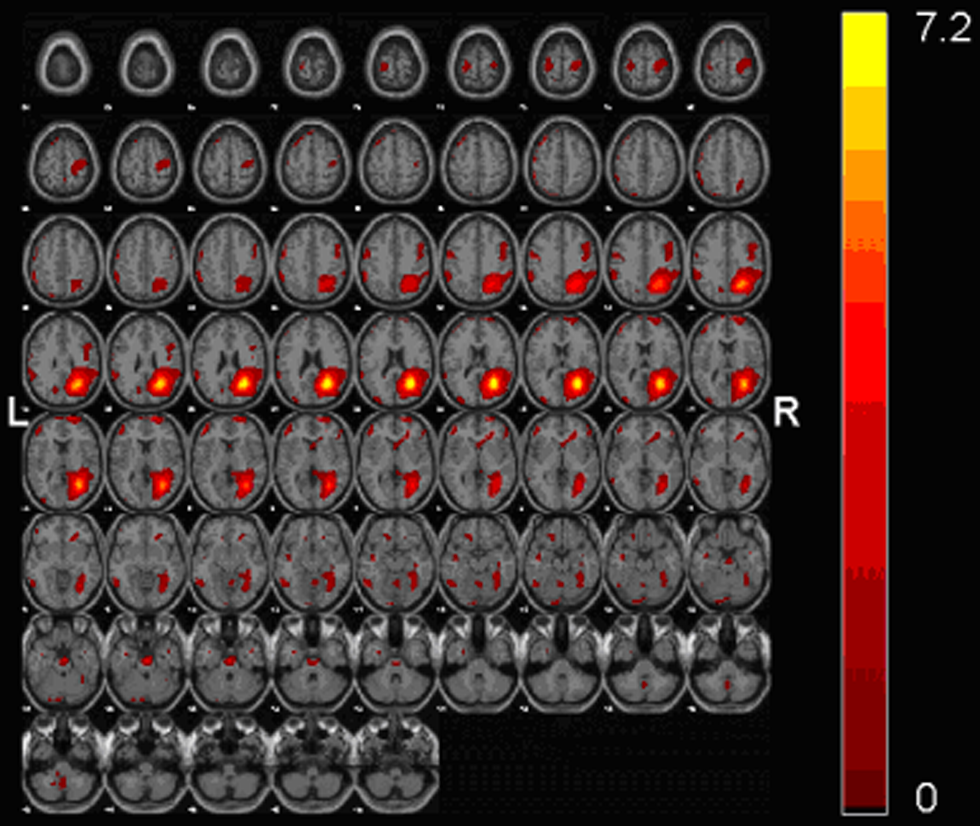

Supplement: Figure S2 — Spatial map of IC2. (TIF) [file pone.0025423.s002.tif]

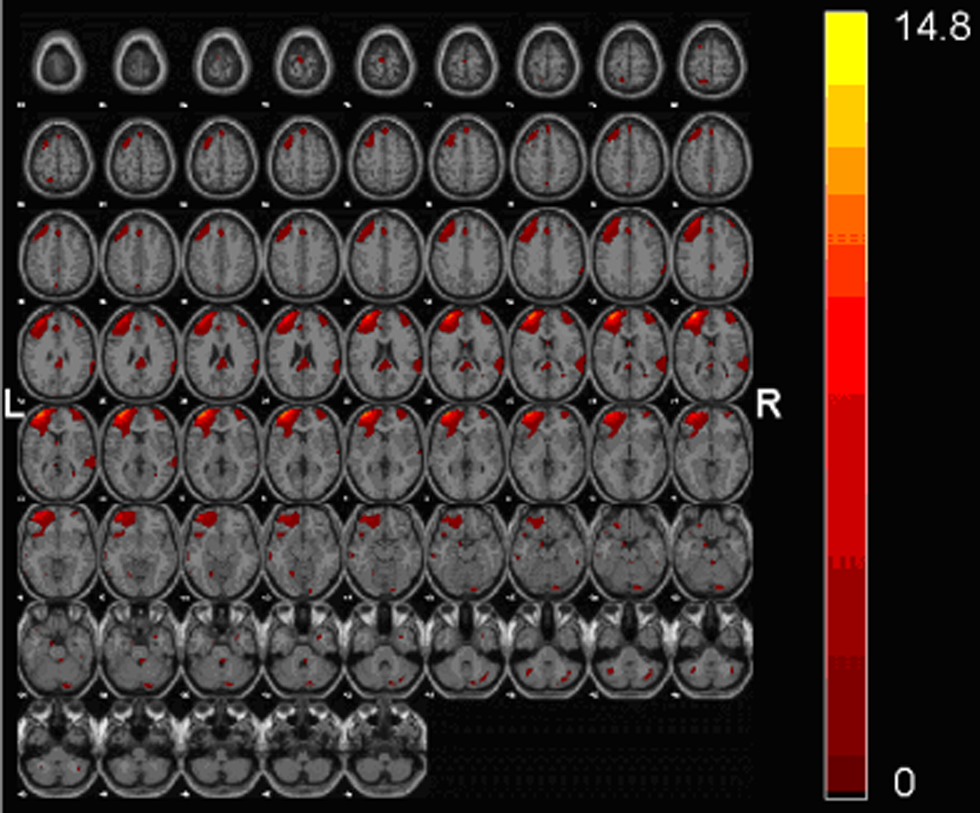

Supplement: Figure S3 — Spatial map of IC3. (TIF) [file pone.0025423.s003.tif]

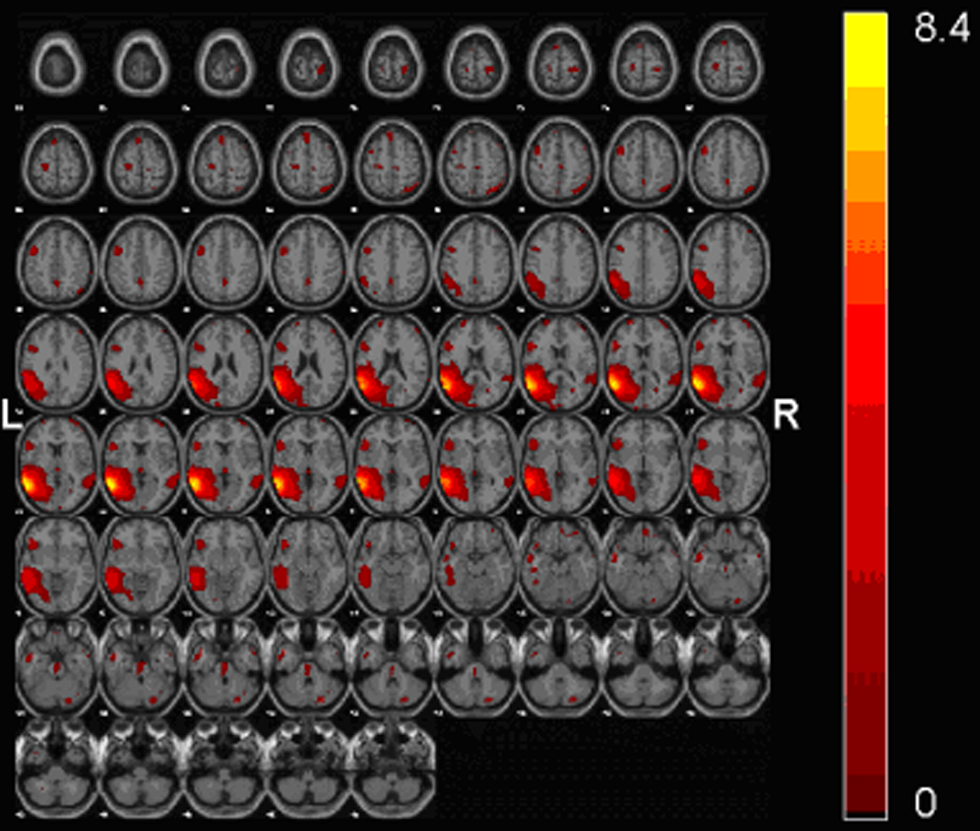

Supplement: Figure S4 — Spatial map of IC4. (TIF) [file pone.0025423.s004.tif]

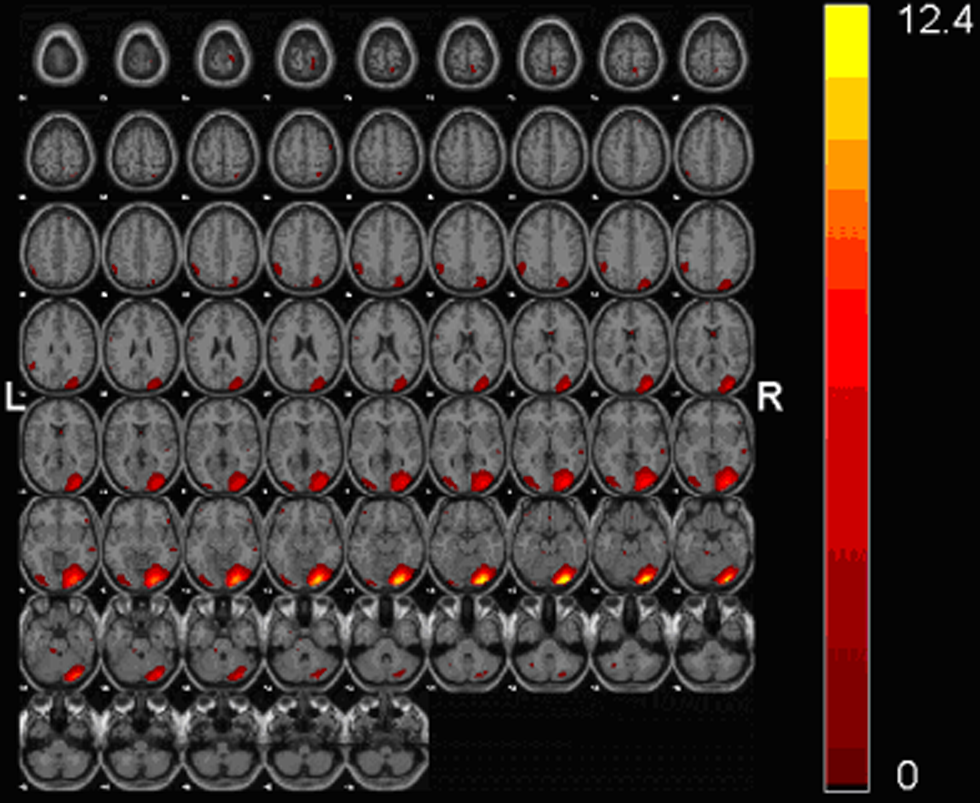

Supplement: Figure S5 — Spatial map of IC5. (TIF) [file pone.0025423.s005.tif]

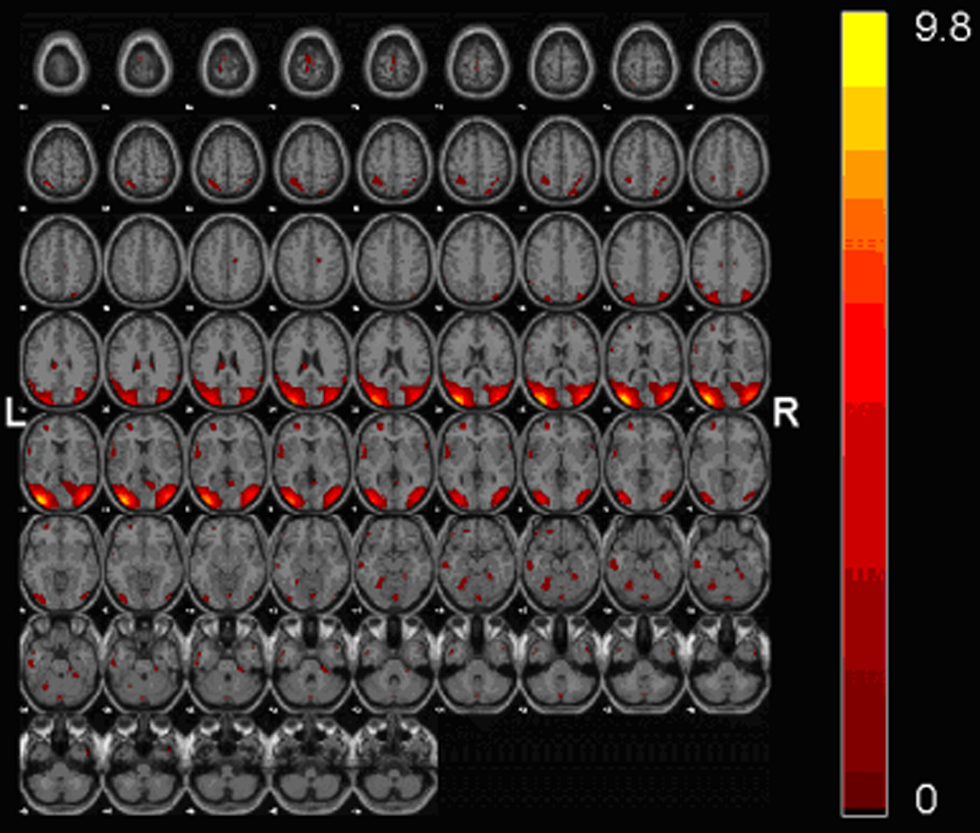

Supplement: Figure S6 — Spatial map of IC6. (TIF) [file pone.0025423.s006.tif]

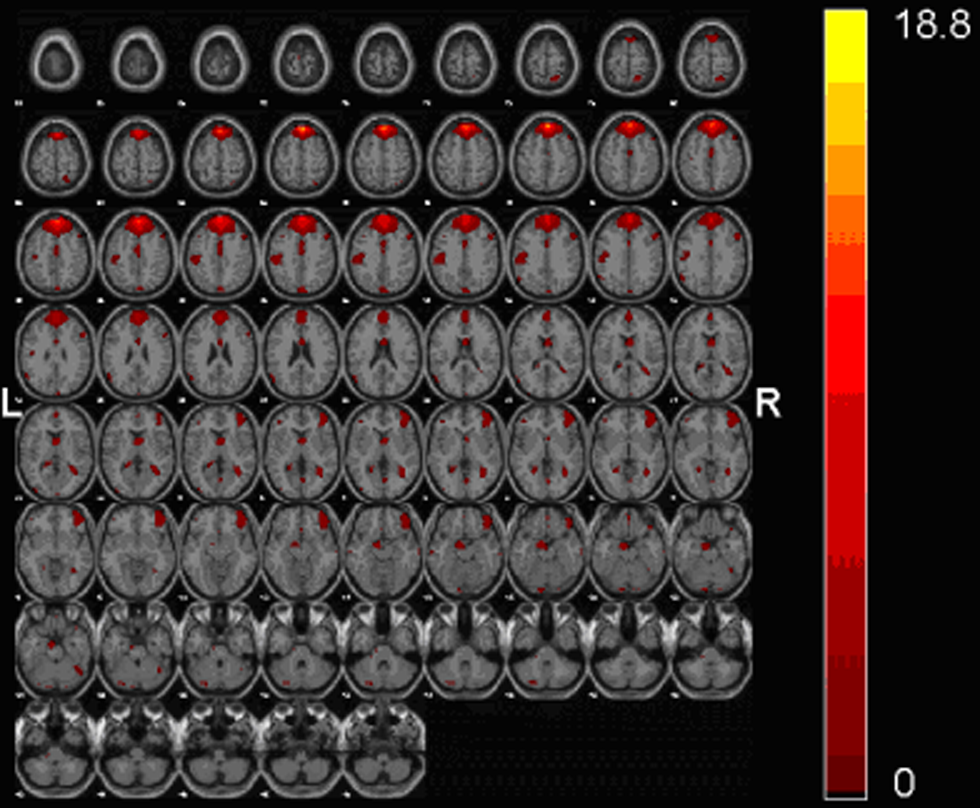

Supplement: Figure S7 — Spatial map of IC7. (TIF) [file pone.0025423.s007.tif]

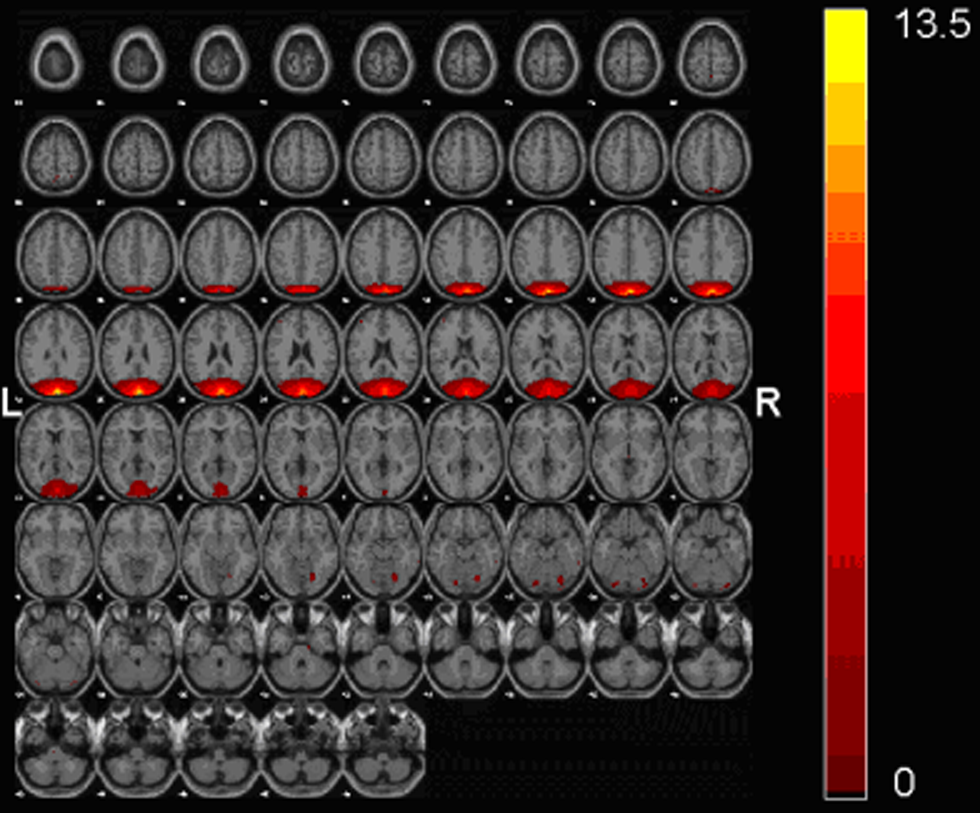

Supplement: Figure S8 — Spatial map of IC8. (TIF) [file pone.0025423.s008.tif]

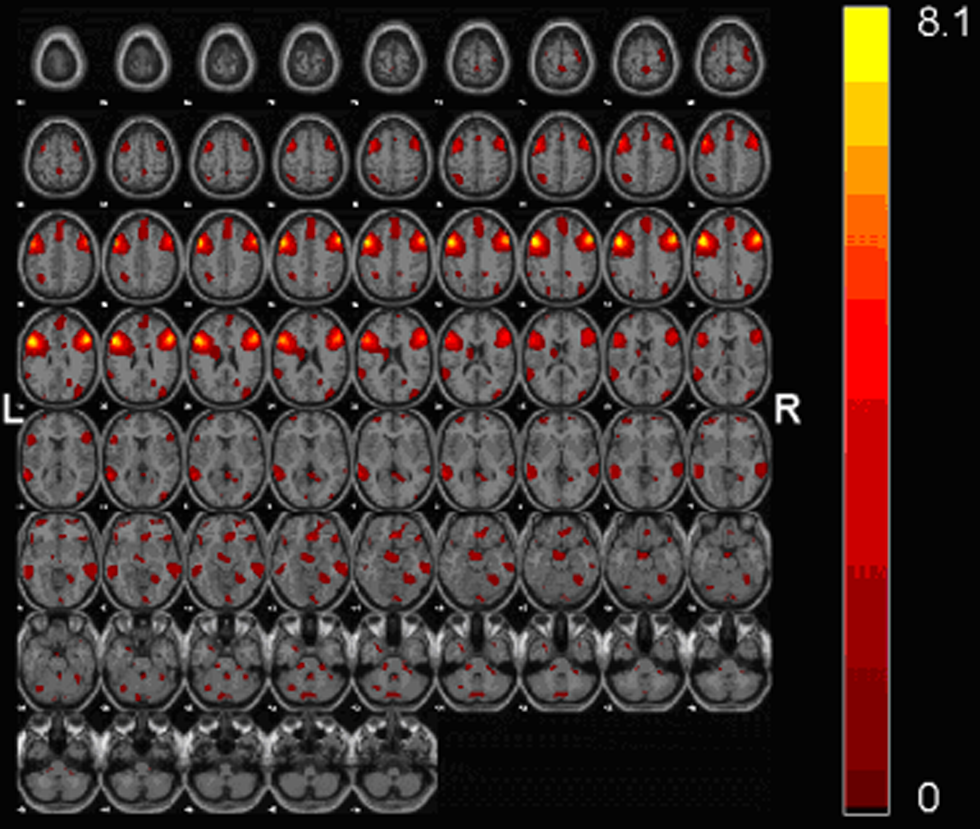

Supplement: Figure S9 — Spatial map of IC9. (TIF) [file pone.0025423.s009.tif]

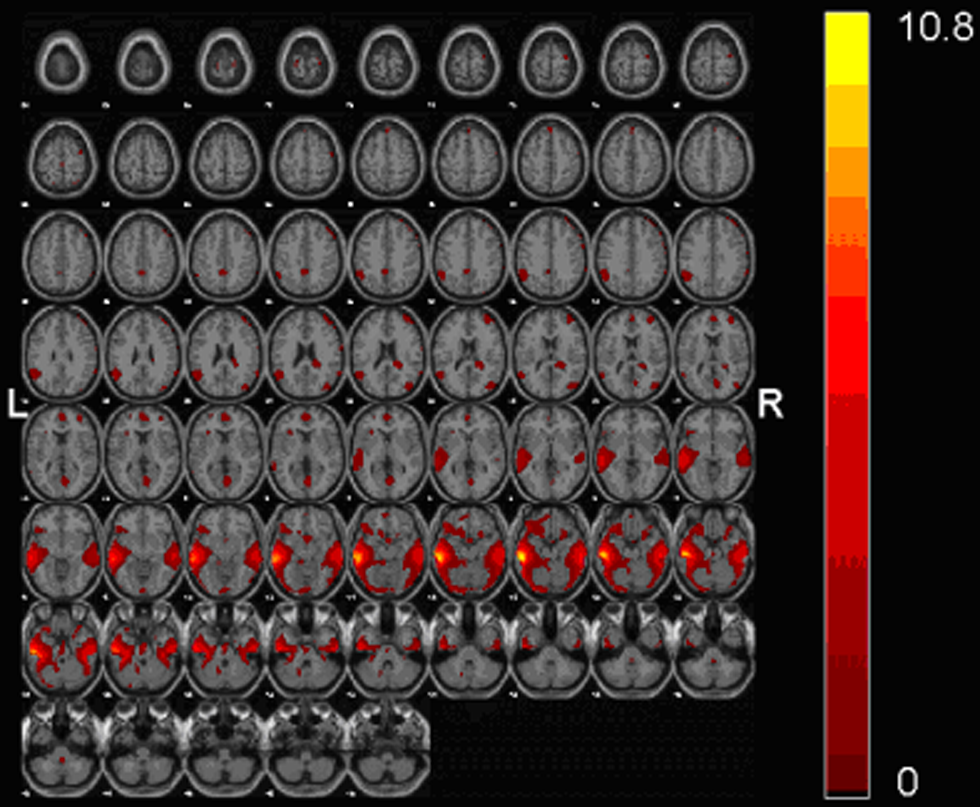

Supplement: Figure S10 — Spatial map of IC10 (TIF) [file pone.0025423.s010.tif]

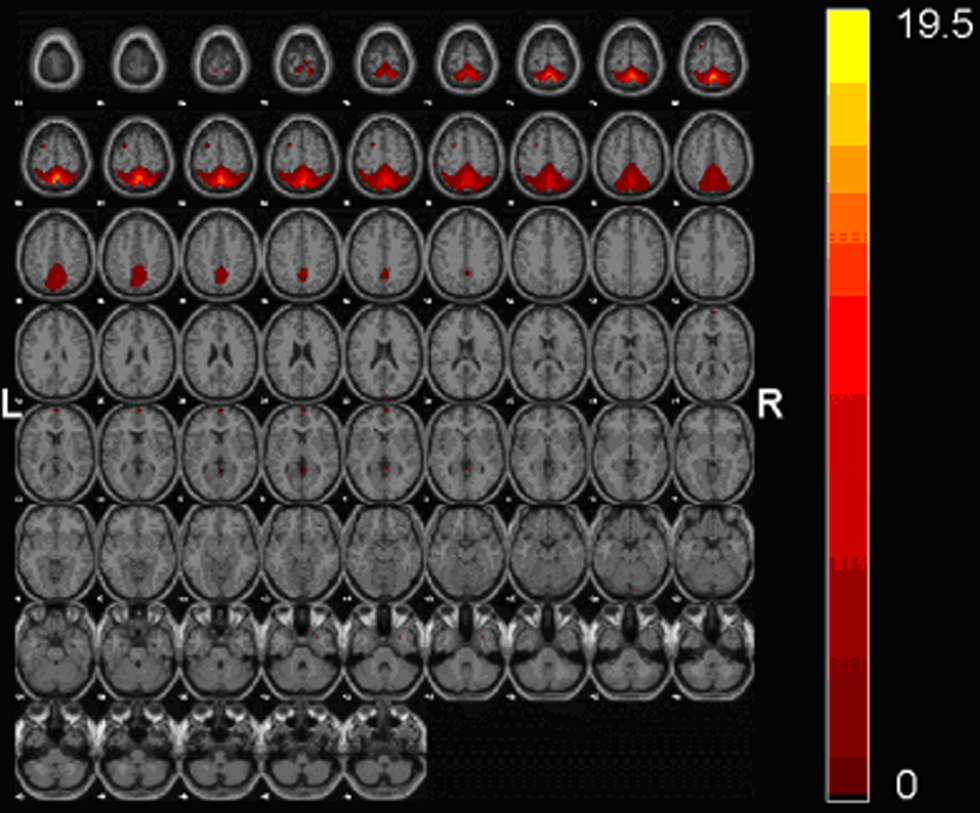

Supplement: Figure S11 — Spatial map of IC11 (TIF) [file pone.0025423.s011.tif]

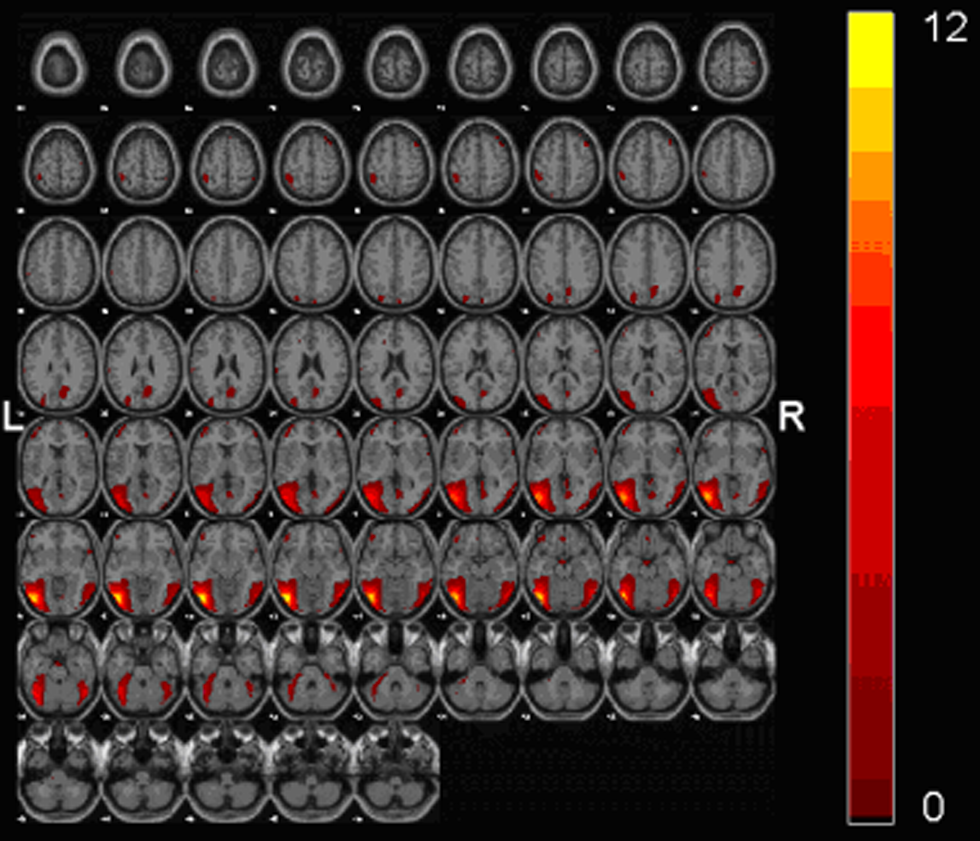

Supplement: Figure S12 — Spatial map of IC12 (TIF) [file pone.0025423.s012.tif]

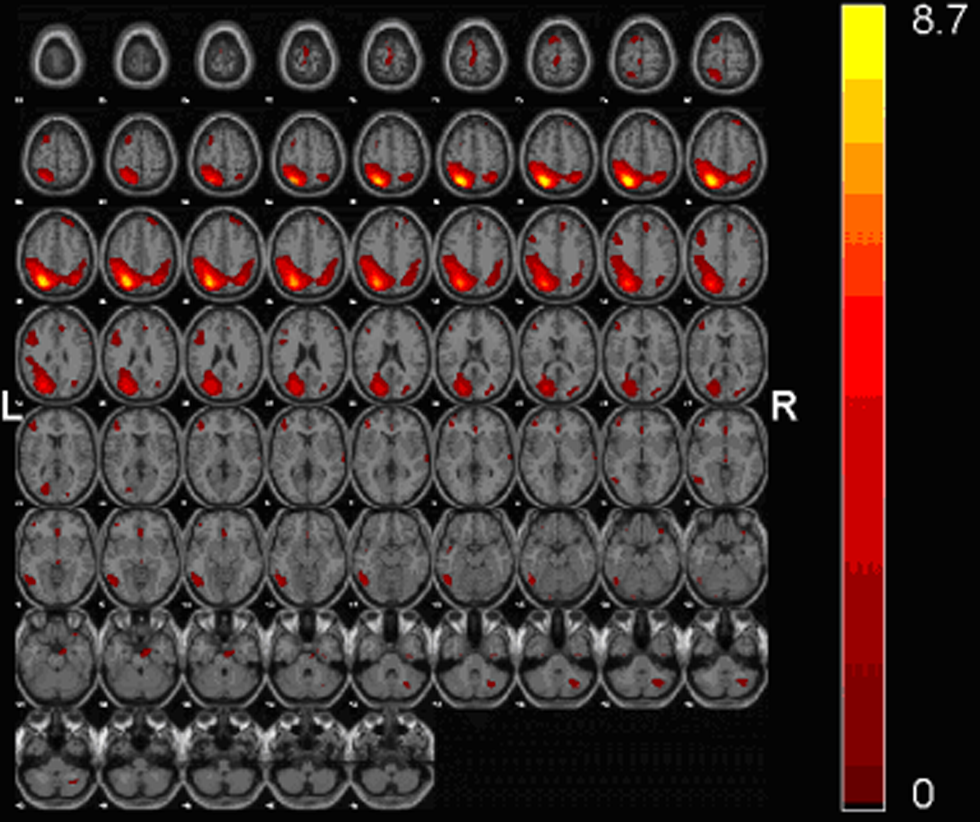

Supplement: Figure S13 — Spatial map of IC13 (TIF) [file pone.0025423.s013.tif]

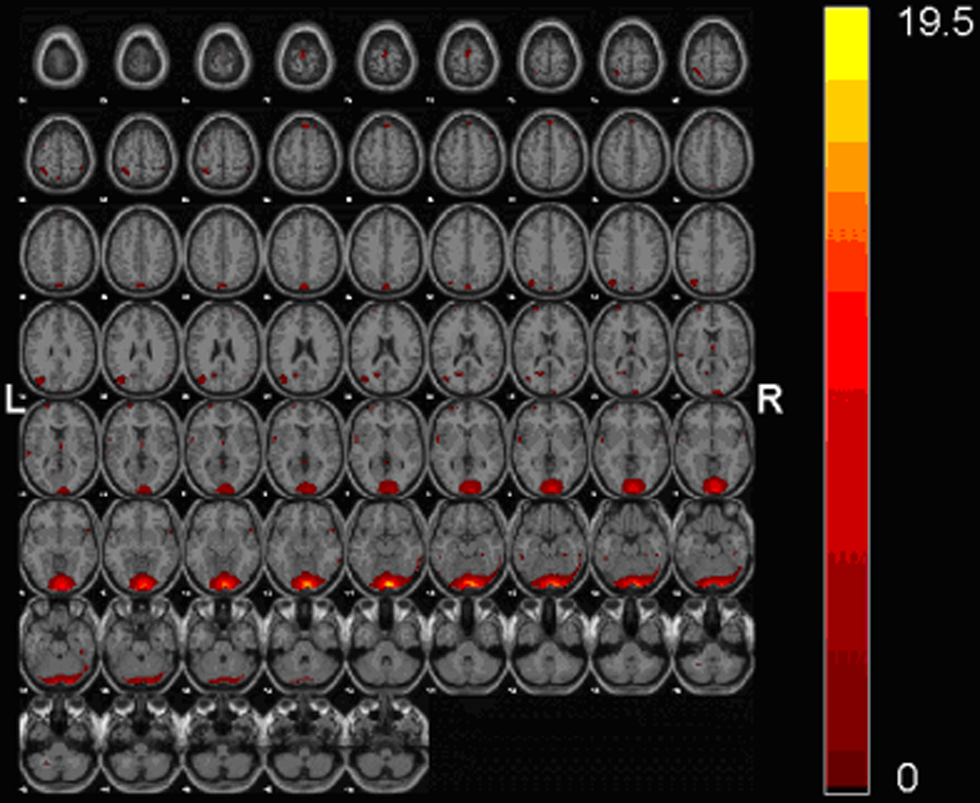

Supplement: Figure S14 — Spatial map of IC14 (TIF) [file pone.0025423.s014.tif]

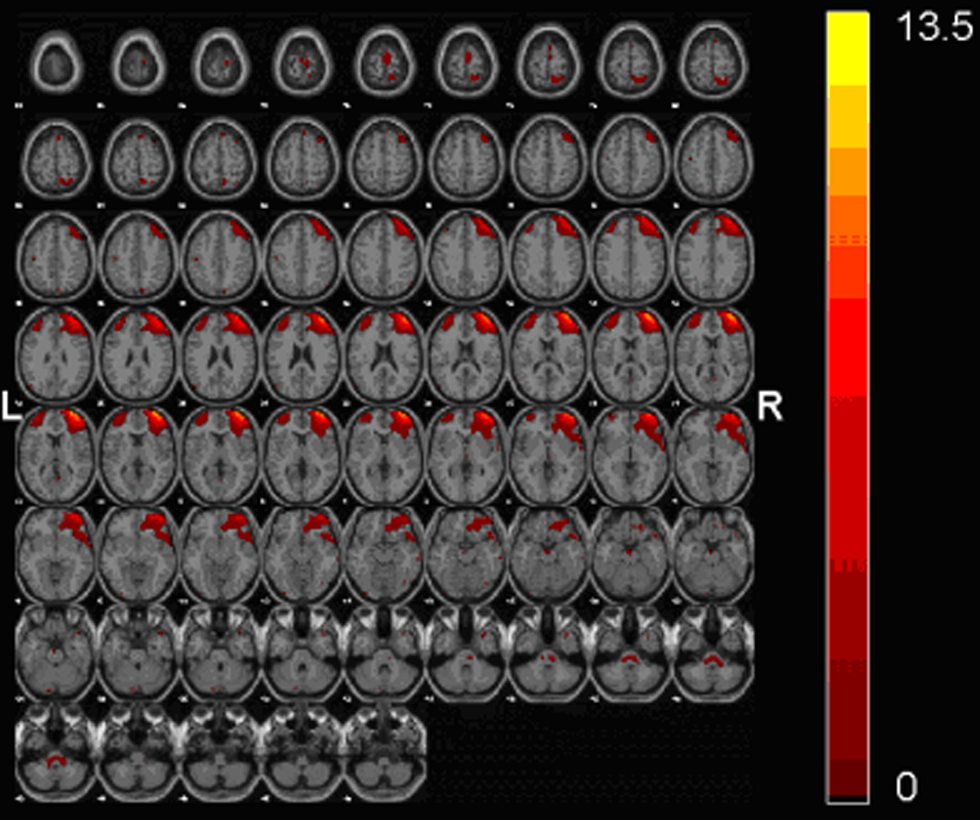

Supplement: Figure S15 — Spatial map of IC15. (TIF) [file pone.0025423.s015.tif]

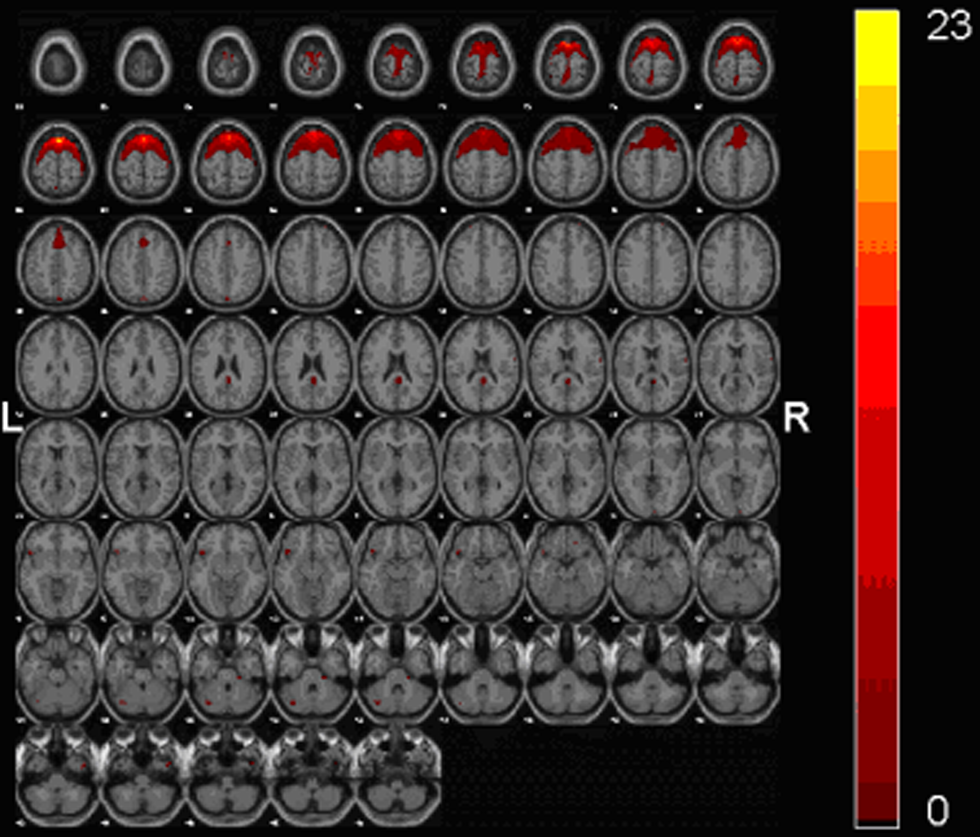

Supplement: Figure S16 — Spatial map of IC16. (TIF) [file pone.0025423.s016.tif]

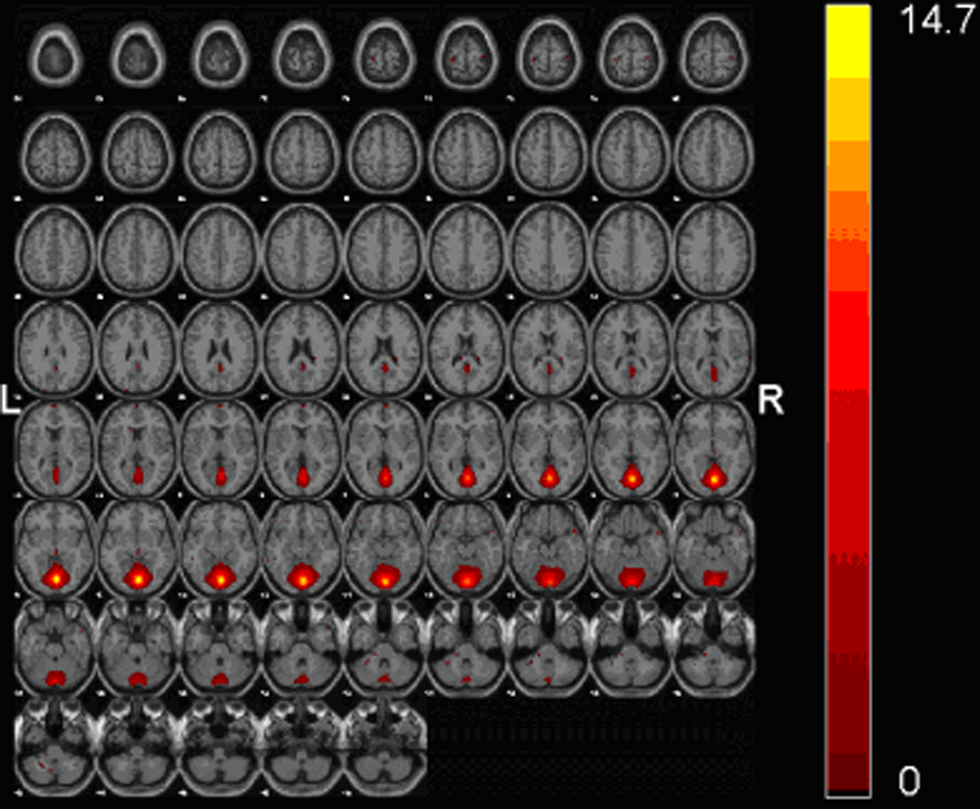

Supplement: Figure S17 — Spatial map of IC17. (TIF) [file pone.0025423.s017.tif]

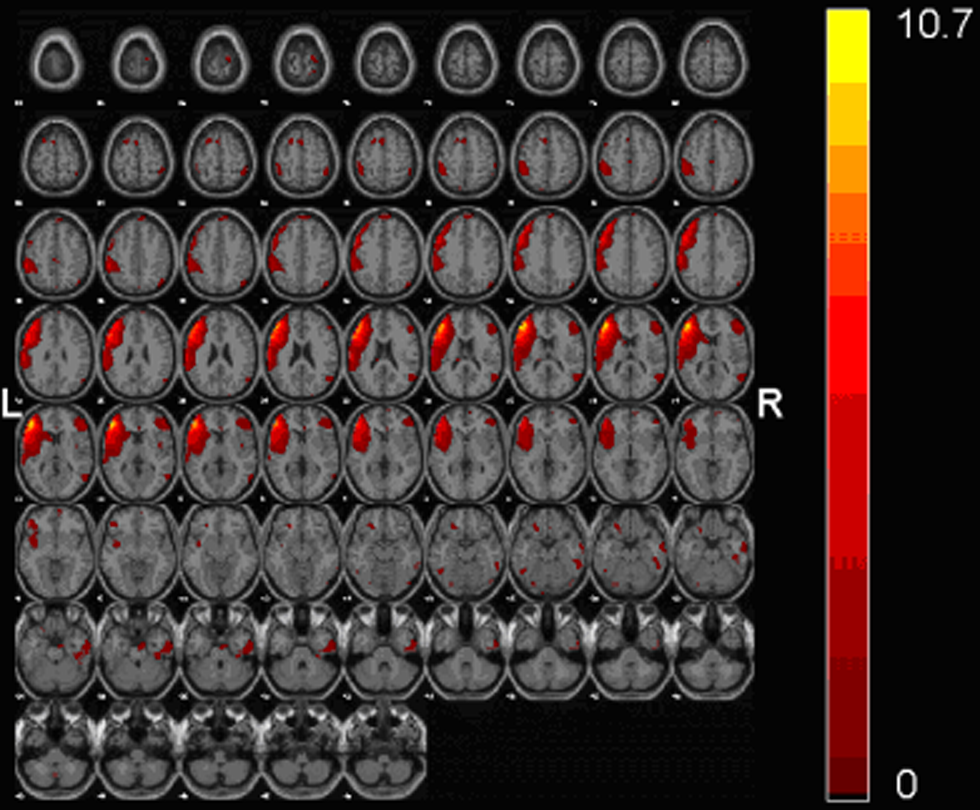

Supplement: Figure S18 — Spatial map of IC18. (TIF) [file pone.0025423.s018.tif]

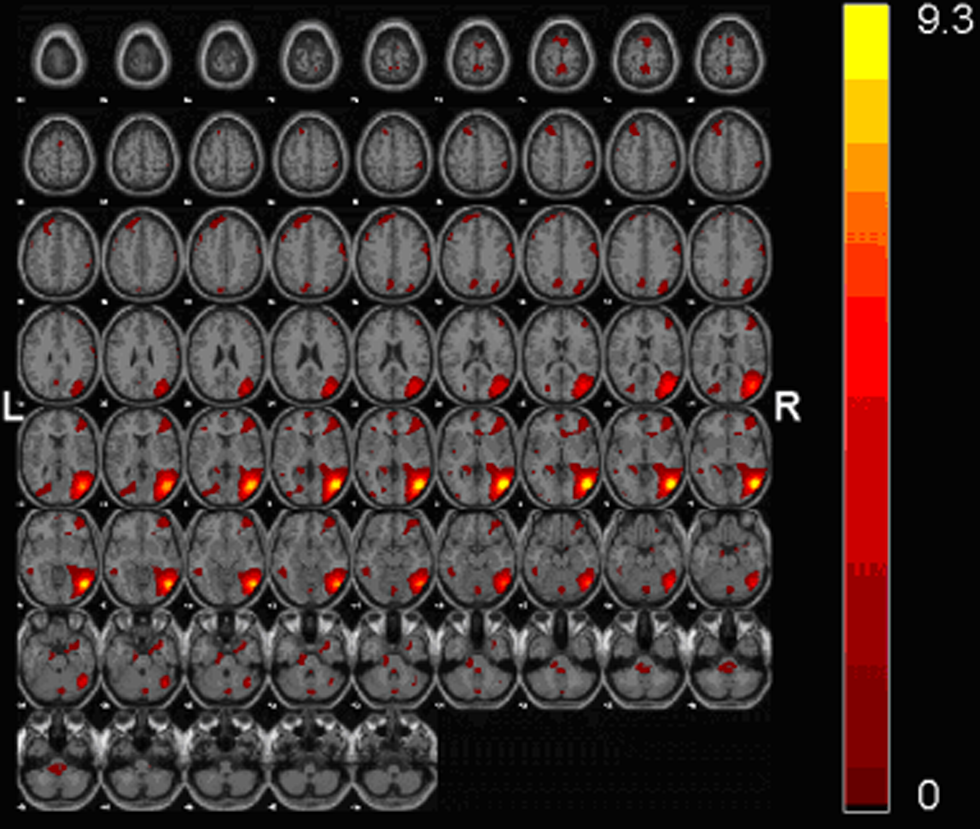

Supplement: Figure S19 — Spatial map of IC19. (TIF) [file pone.0025423.s019.tif]

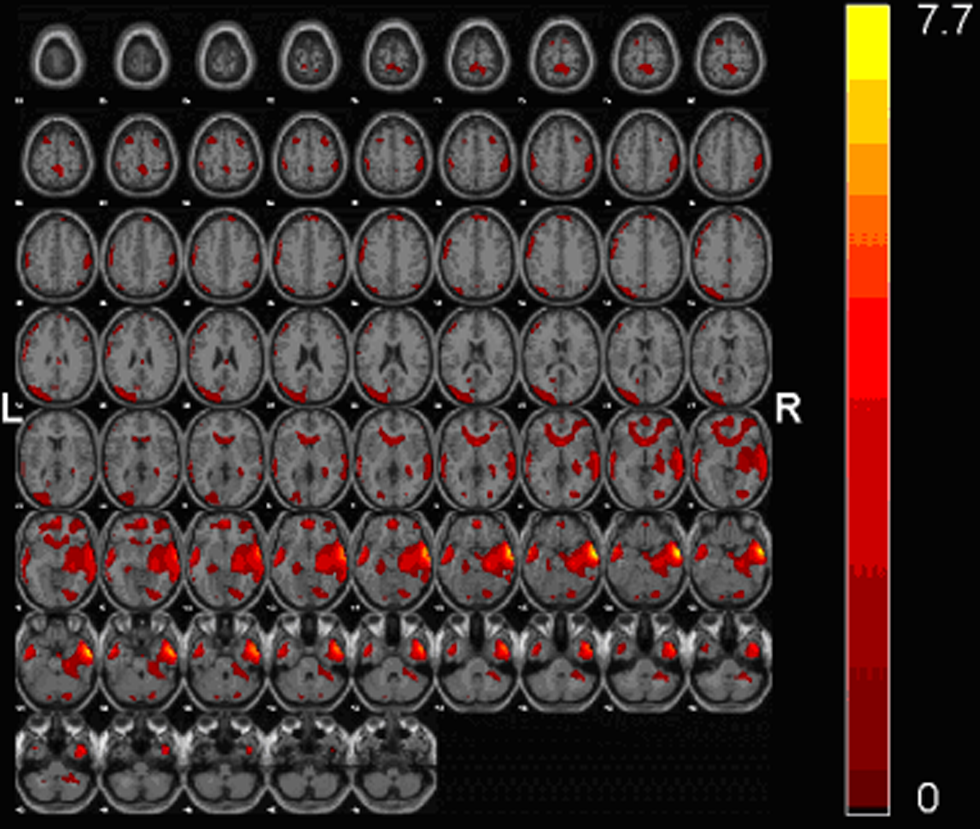

Supplement: Figure S20 — Spatial map of IC20. (TIF) [file pone.0025423.s020.tif]

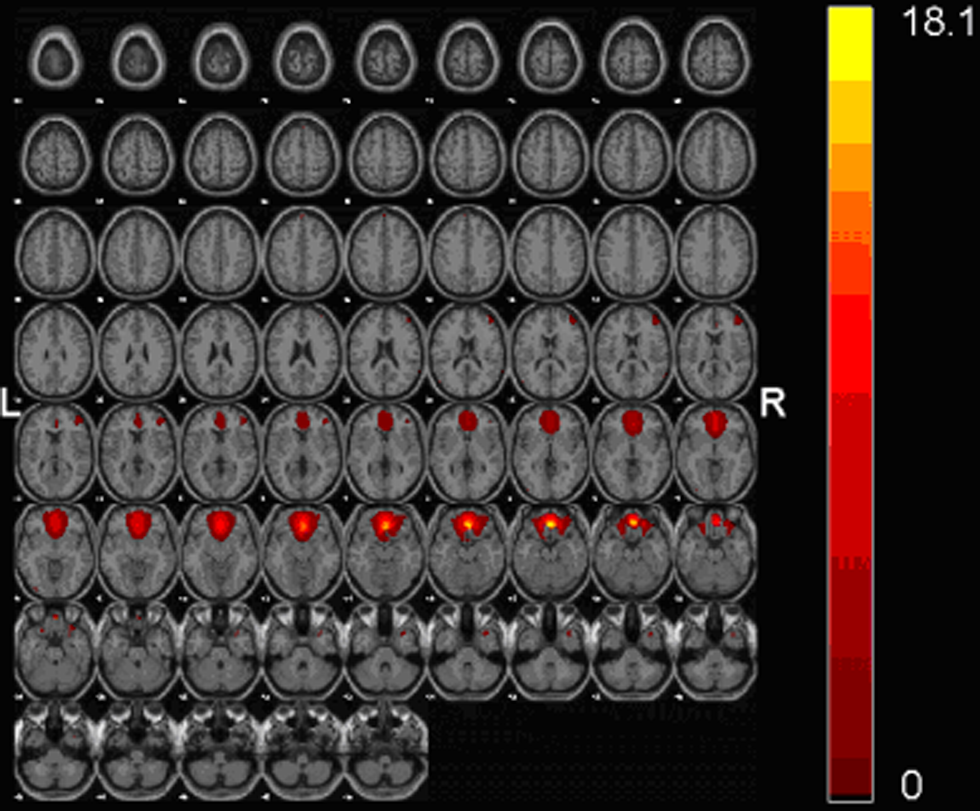

Supplement: Figure S21 — Spatial map of IC21. (TIF) [file pone.0025423.s021.tif]

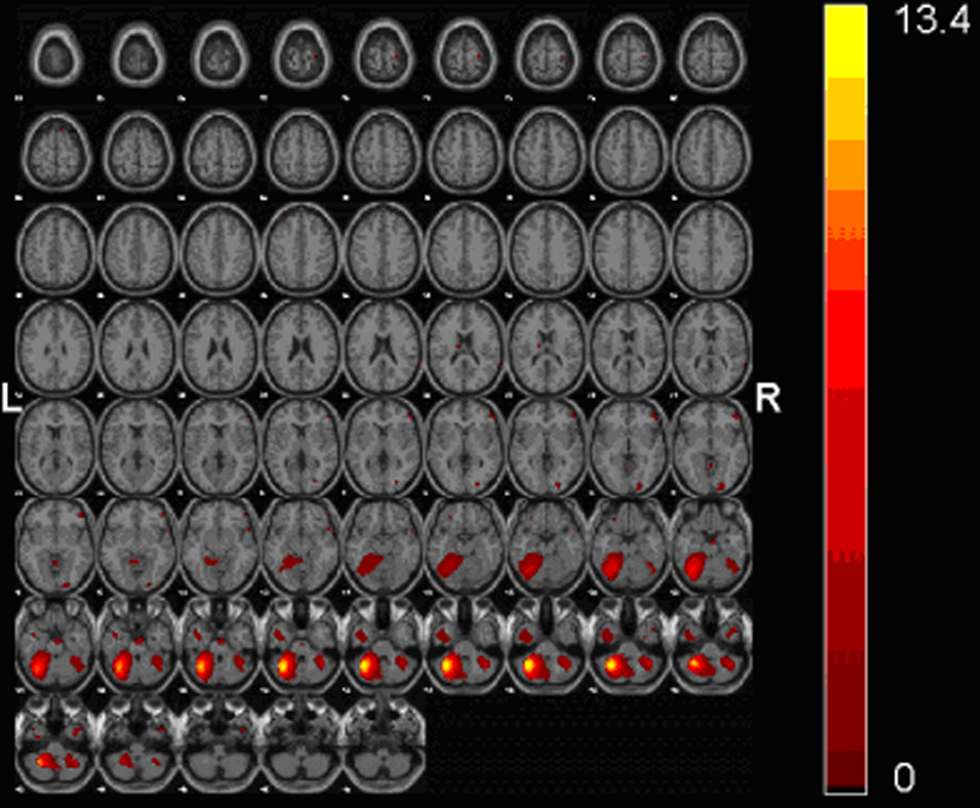

Supplement: Figure S22 — Spatial map of IC22. (TIF) [file pone.0025423.s022.tif]

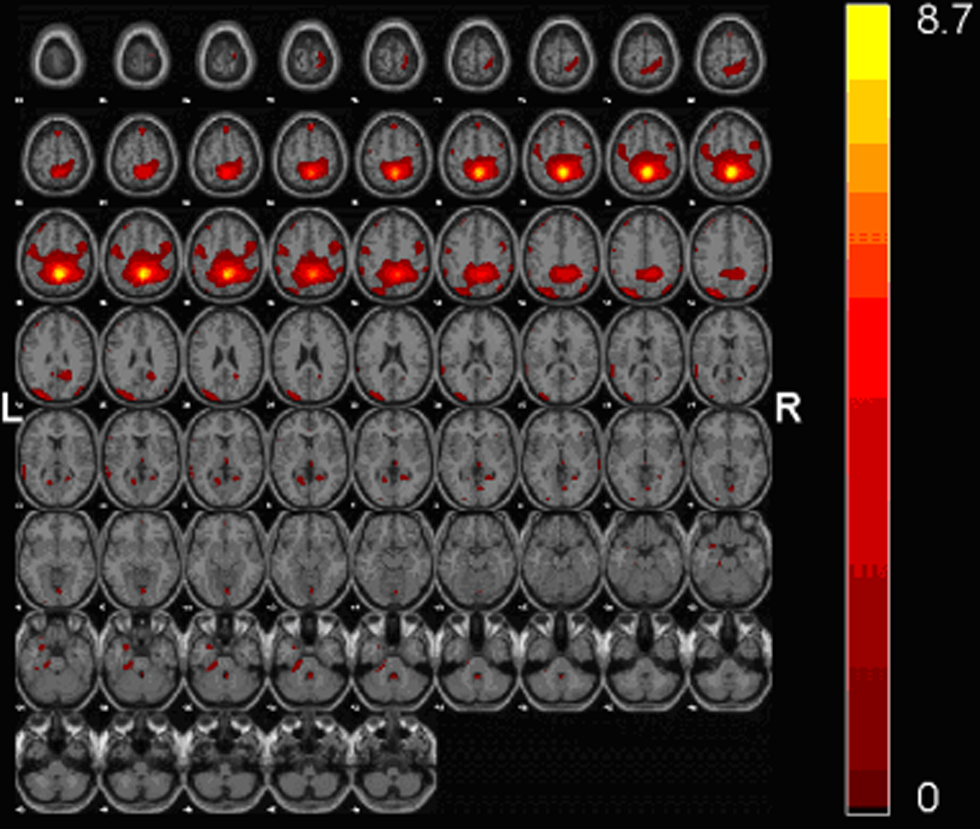

Supplement: Figure S23 — Spatial map of IC23. (TIF) [file pone.0025423.s023.tif]

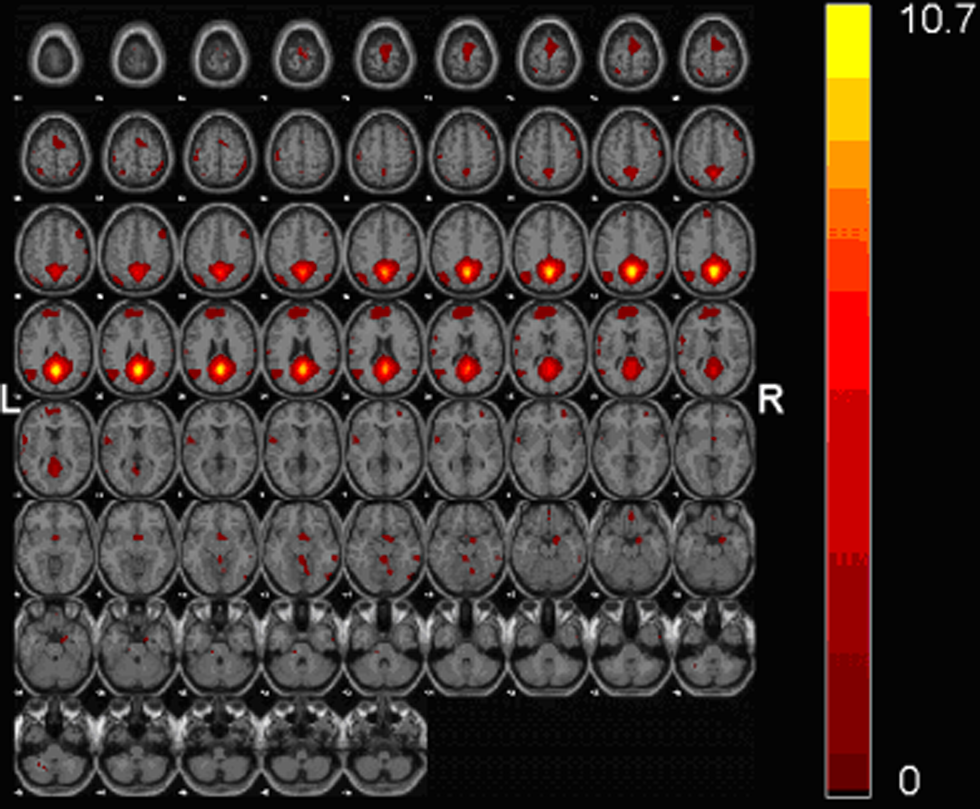

Supplement: Figure S24 — Spatial map of IC24. (TIF) [file pone.0025423.s024.tif]

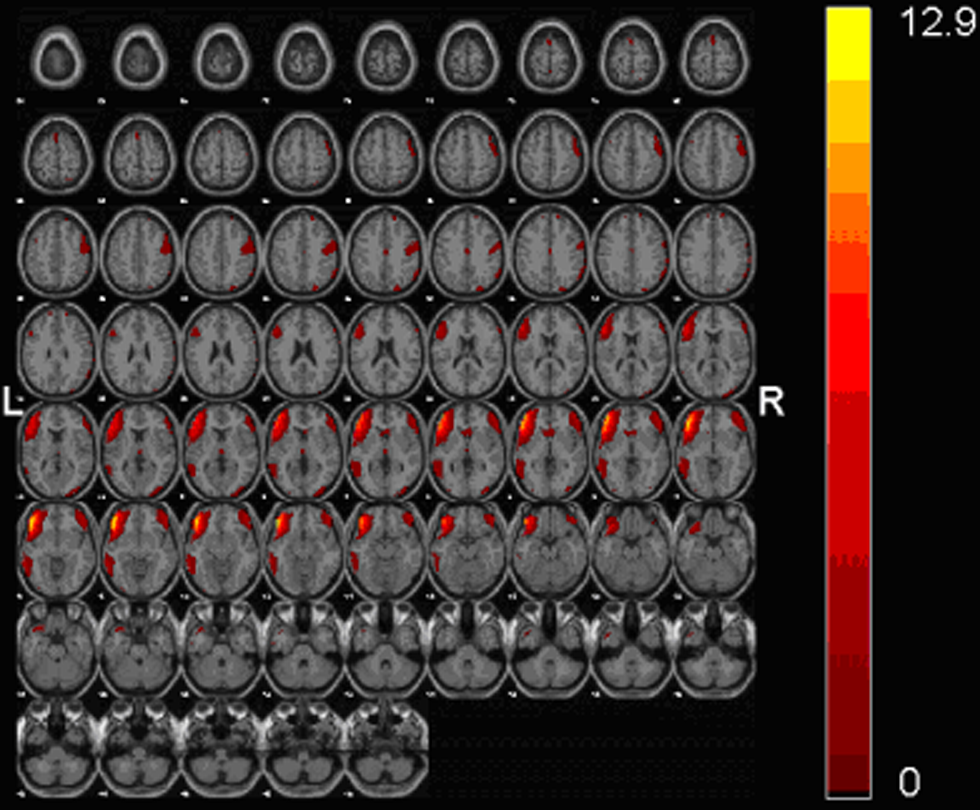

Supplement: Figure S25 — Spatial map of IC25. (TIF) [file pone.0025423.s025.tif]

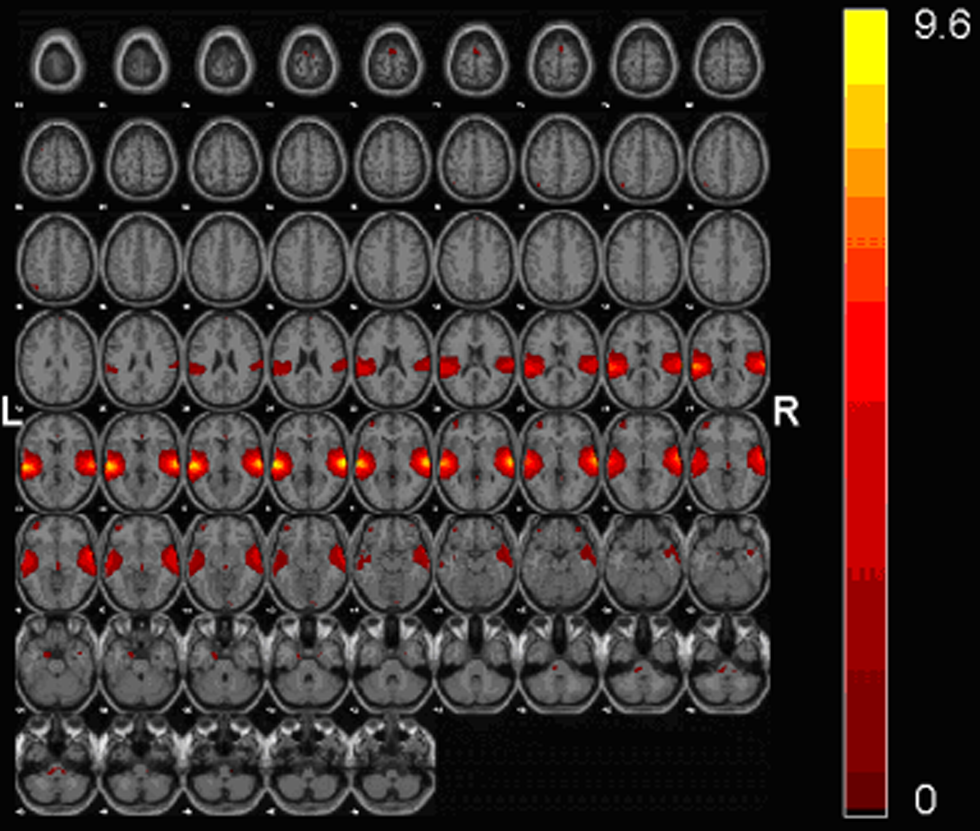

Supplement: Figure S26 — Spatial map of IC26. (TIF) [file pone.0025423.s026.tif]

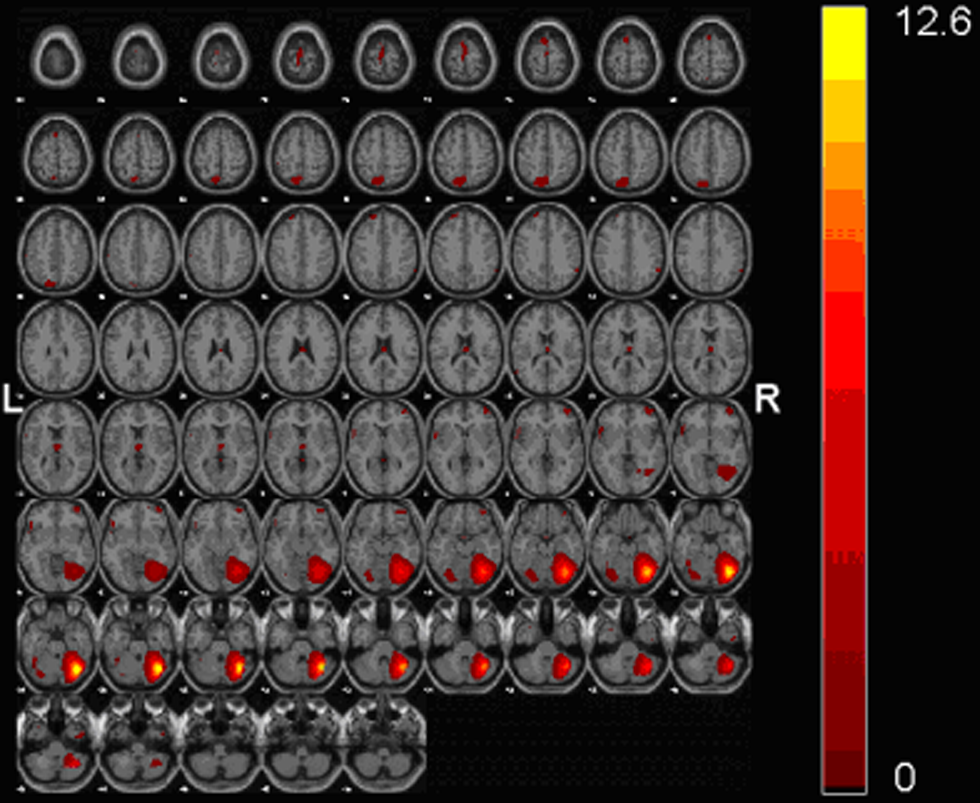

Supplement: Figure S27 — Spatial map of IC27. (TIF) [file pone.0025423.s027.tif]

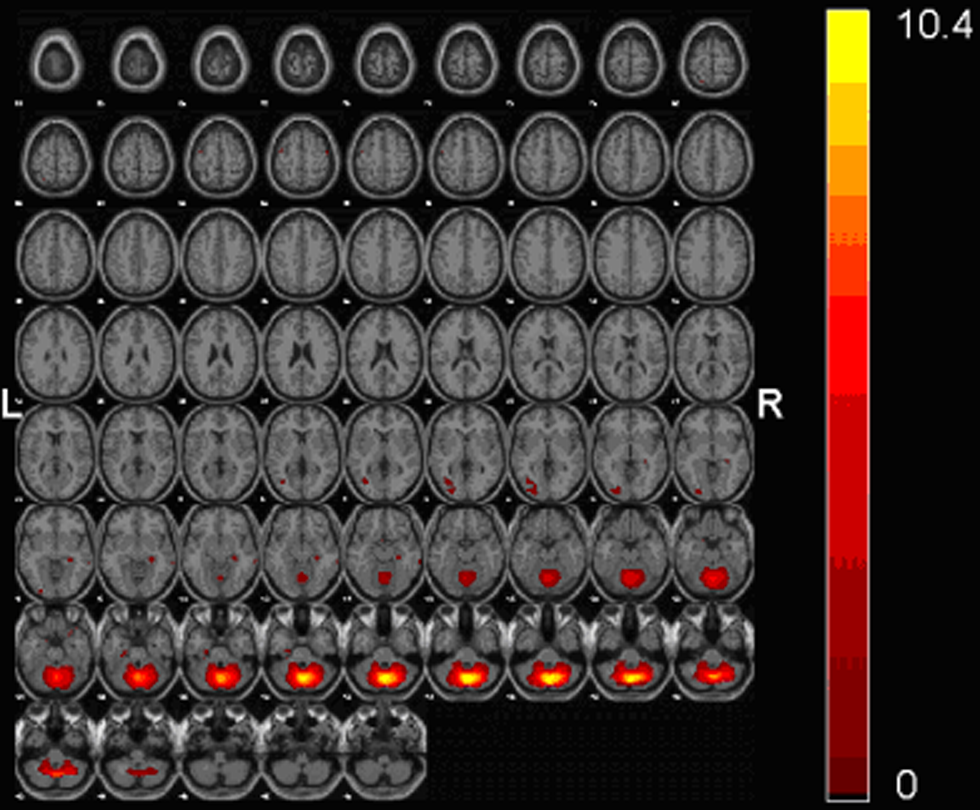

Supplement: Figure S28 — Spatial map of IC28. (TIF) [file pone.0025423.s028.tif]

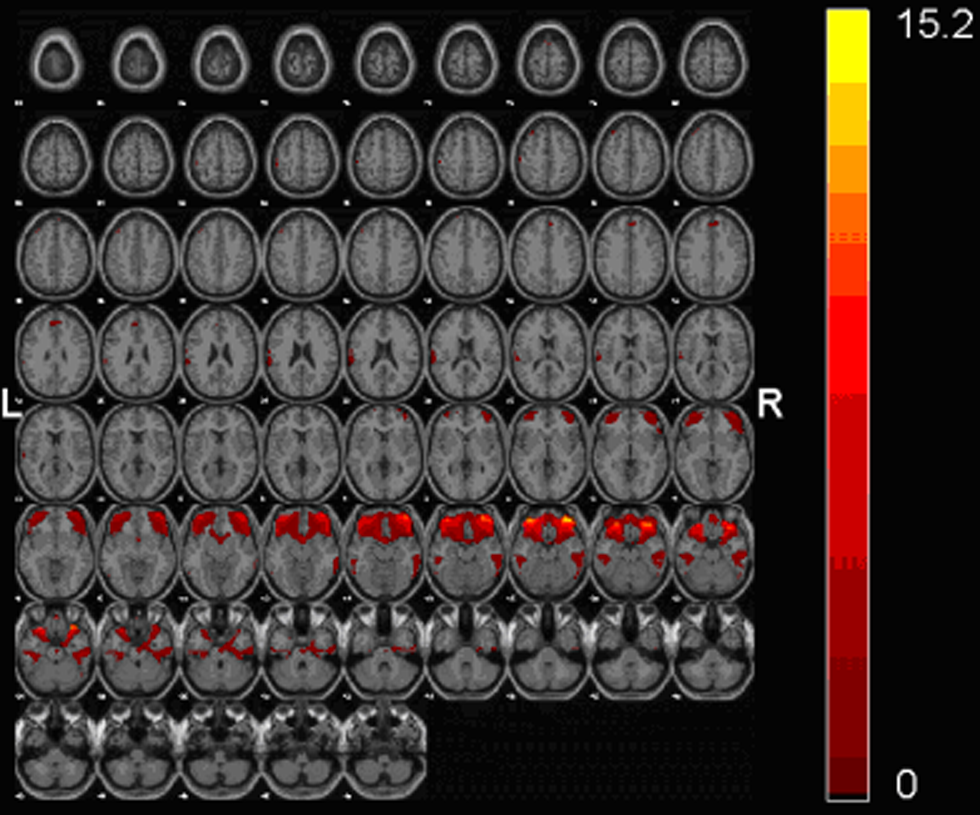

Supplement: Figure S29 — Spatial map of IC29. (TIF) [file pone.0025423.s029.tif]

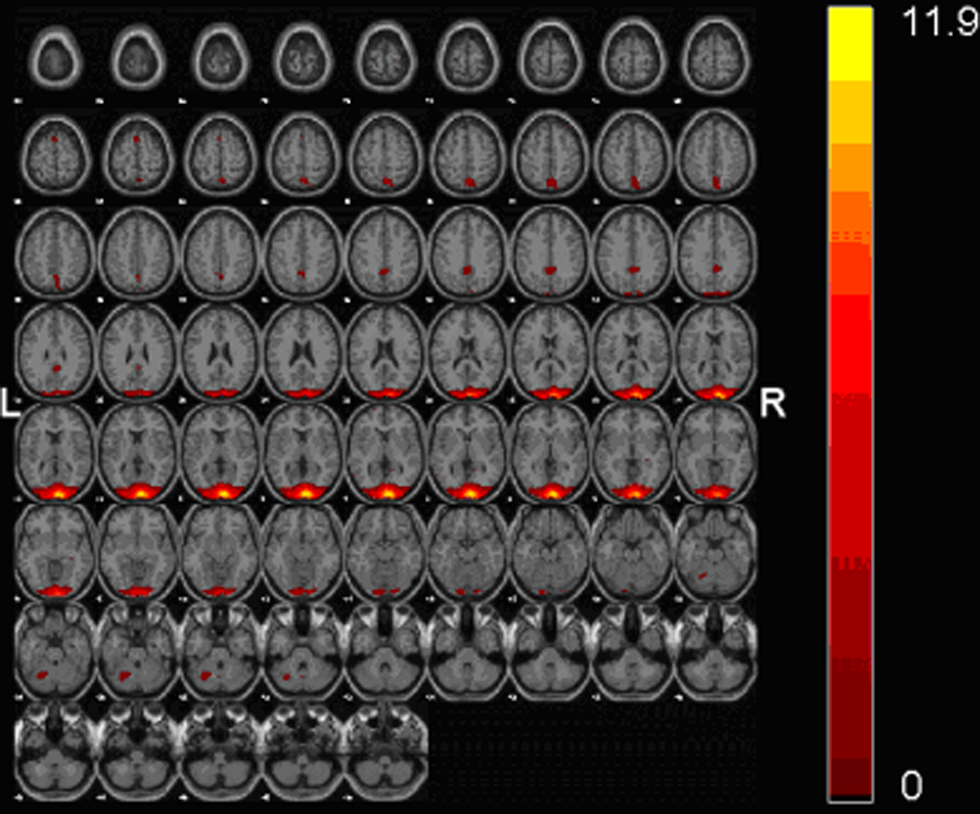

Supplement: Figure S30 — Spatial map of IC30. (TIF) [file pone.0025423.s030.tif]

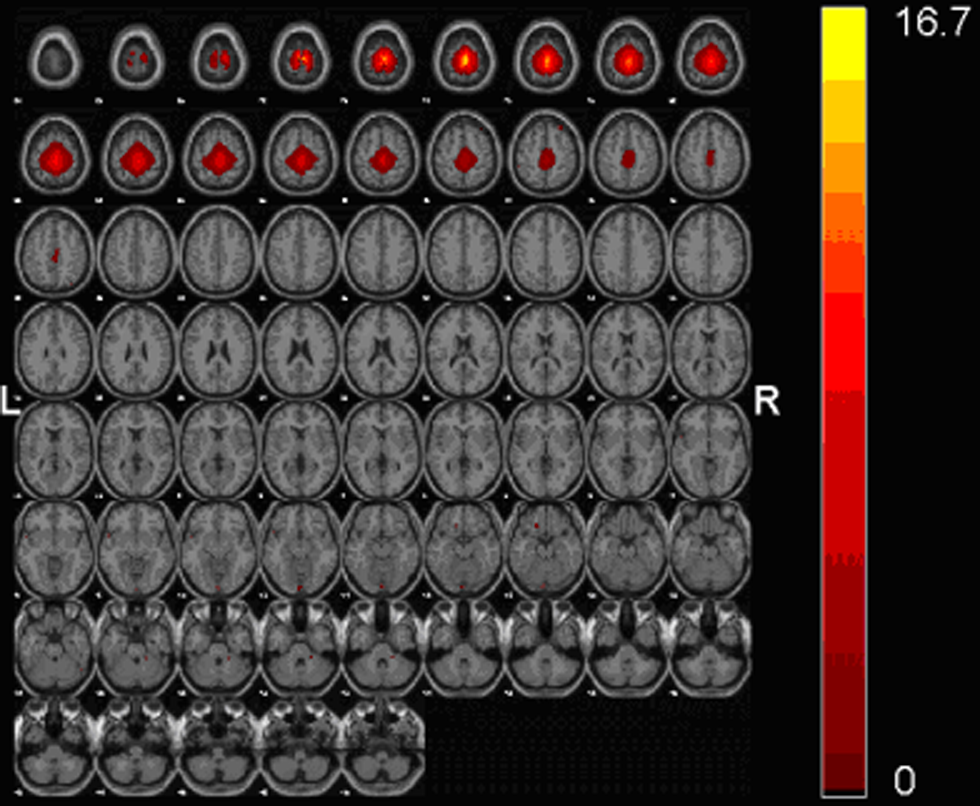

Supplement: Figure S31 — Spatial map of IC31. (TIF) [file pone.0025423.s031.tif]

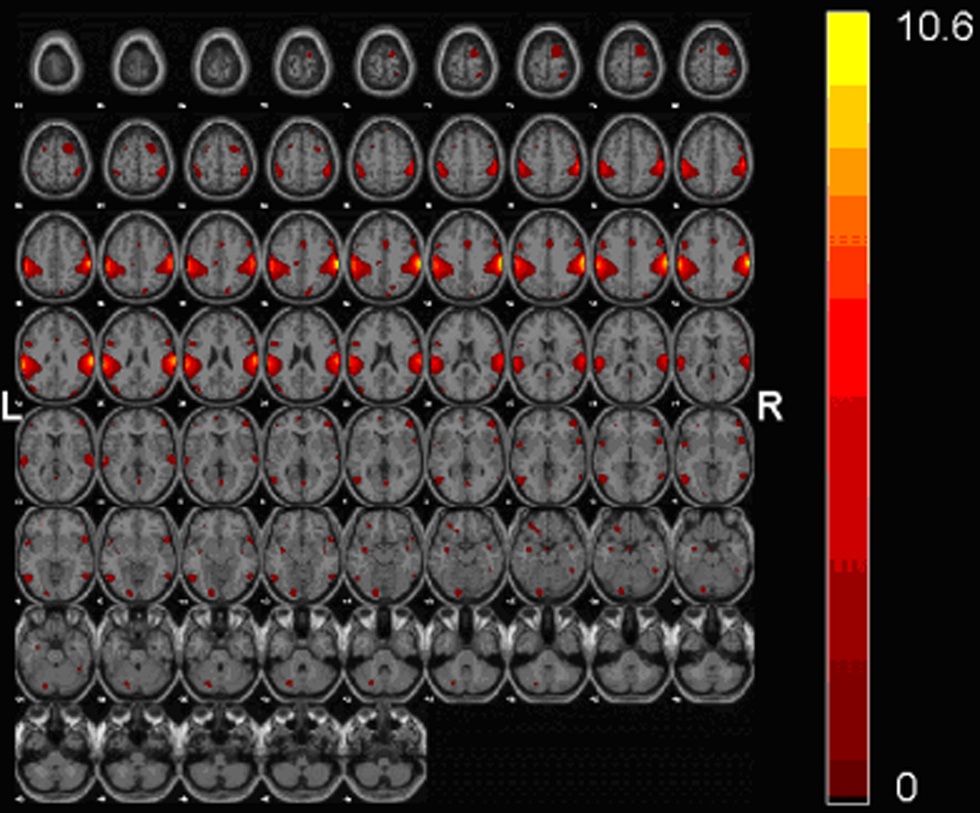

Supplement: Figure S32 — Spatial map of IC32. (TIF) [file pone.0025423.s032.tif]

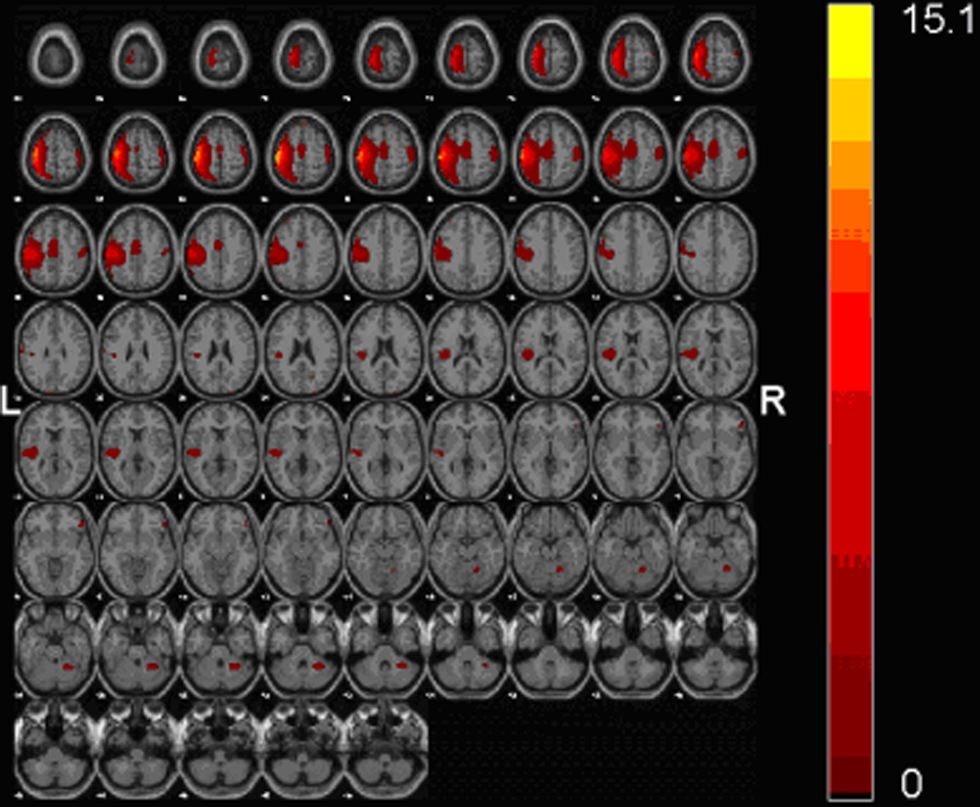

Supplement: Figure S33 — Spatial map of IC33. (TIF) [file pone.0025423.s033.tif]

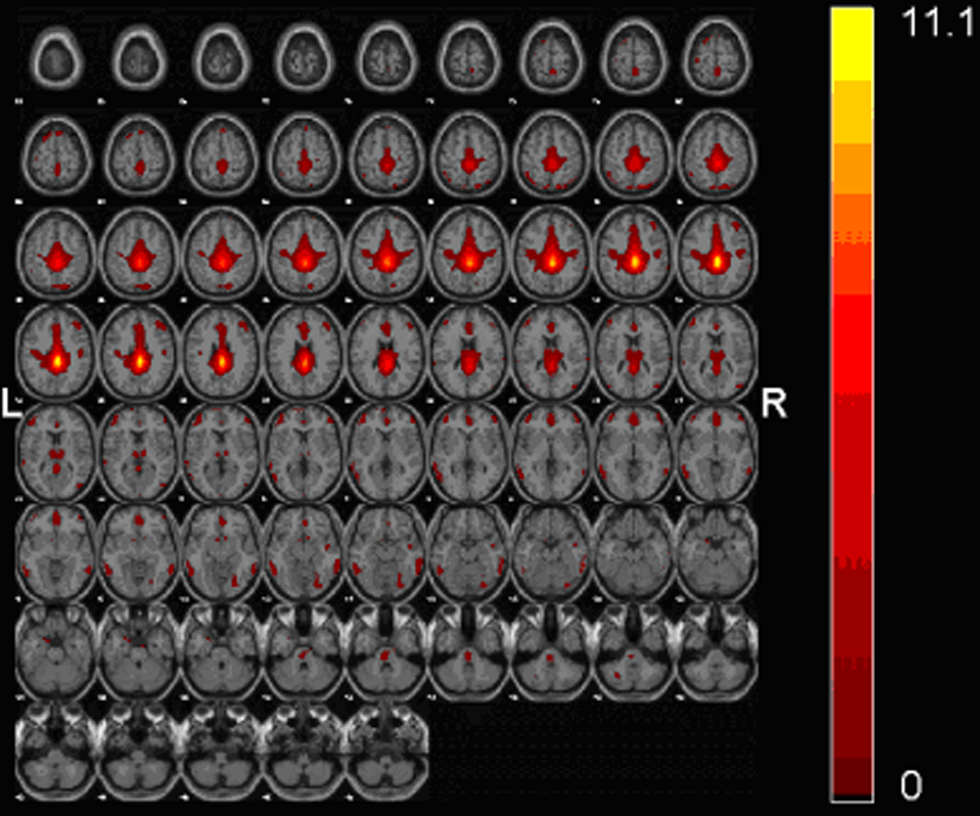

Supplement: Figure S34 — Spatial map of IC34. (TIF) [file pone.0025423.s034.tif]

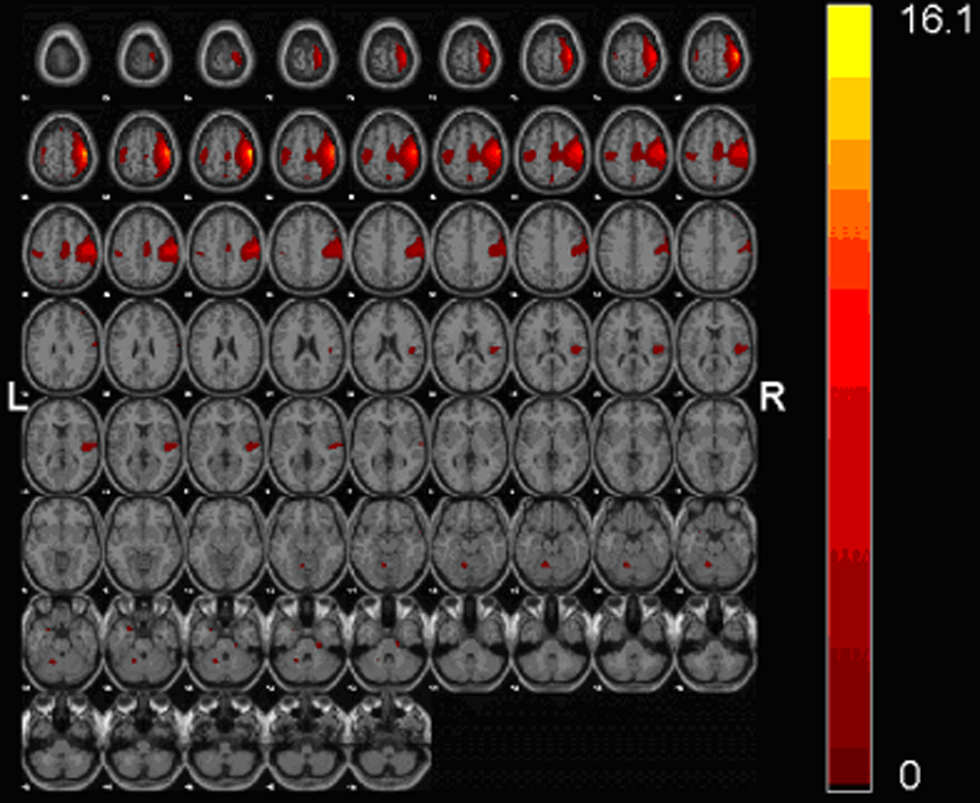

Supplement: Figure S35 — Spatial map of IC35. (TIF) [file pone.0025423.s035.tif]

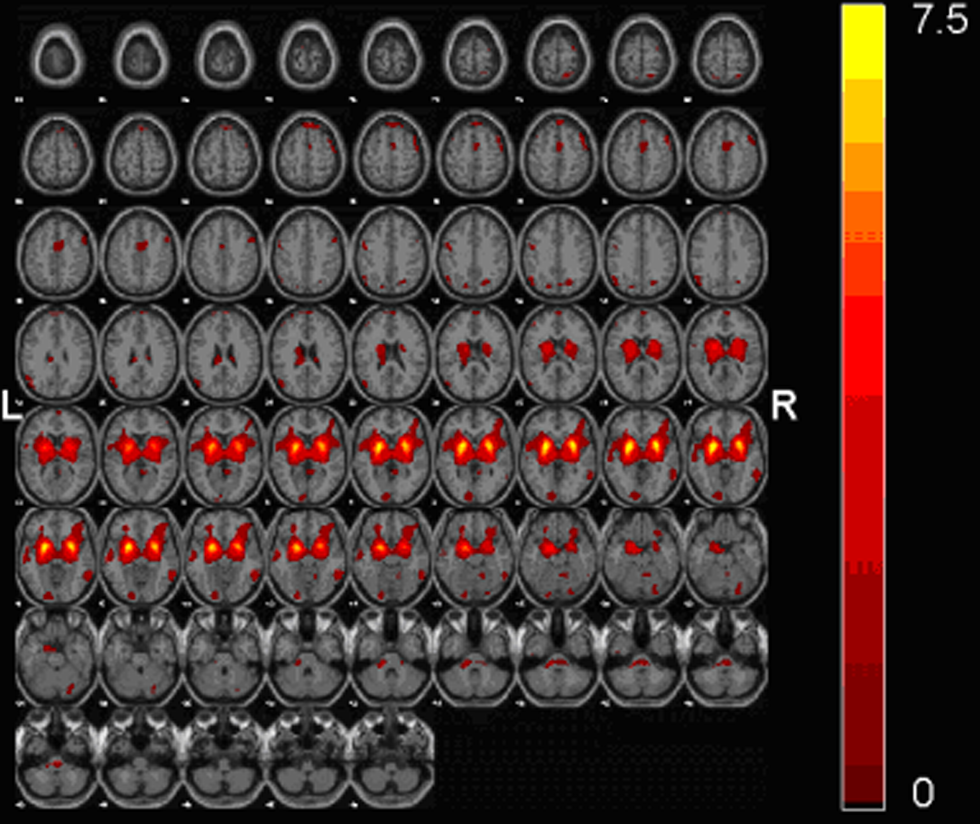

Supplement: Figure S36 — Spatial map of IC36. (TIF) [file pone.0025423.s036.tif]

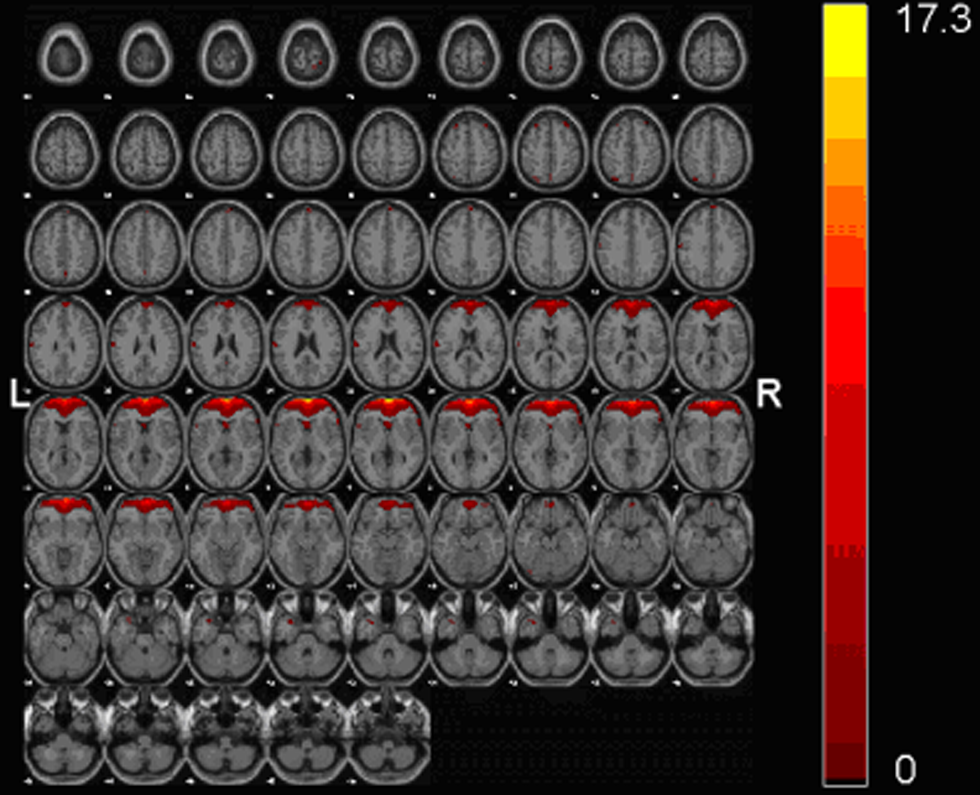

Supplement: Figure S37 — Spatial map of IC37. (TIF) [file pone.0025423.s037.tif]

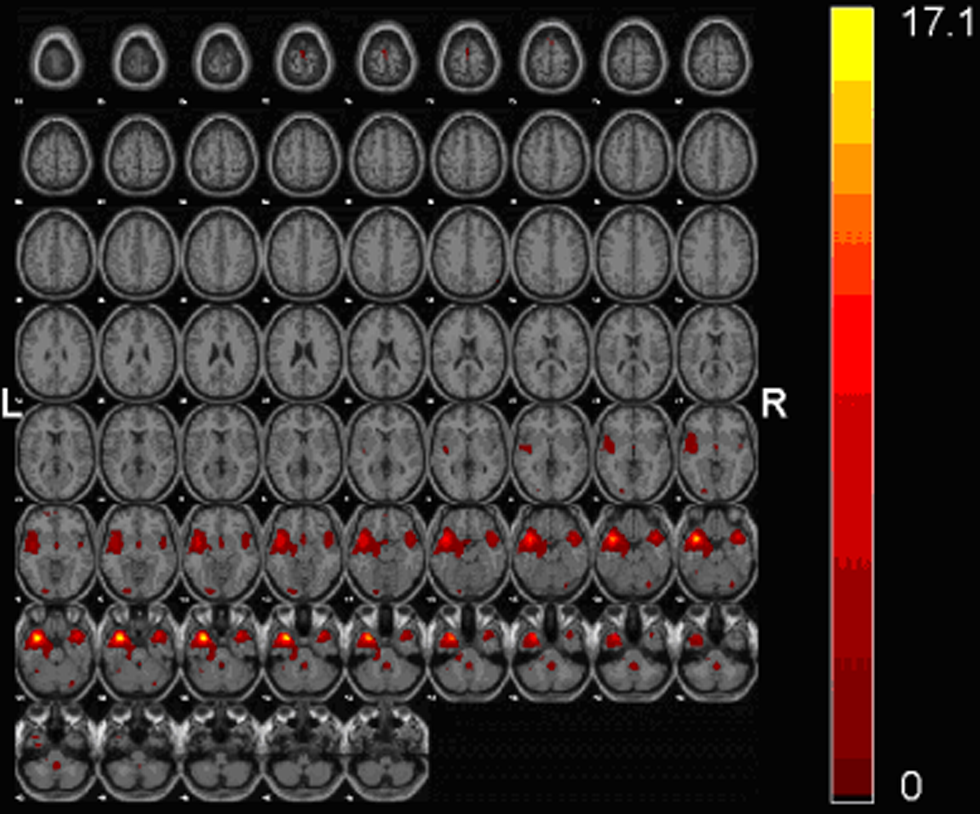

Supplement: Figure S38 — Spatial map of IC38. (TIF) [file pone.0025423.s038.tif]

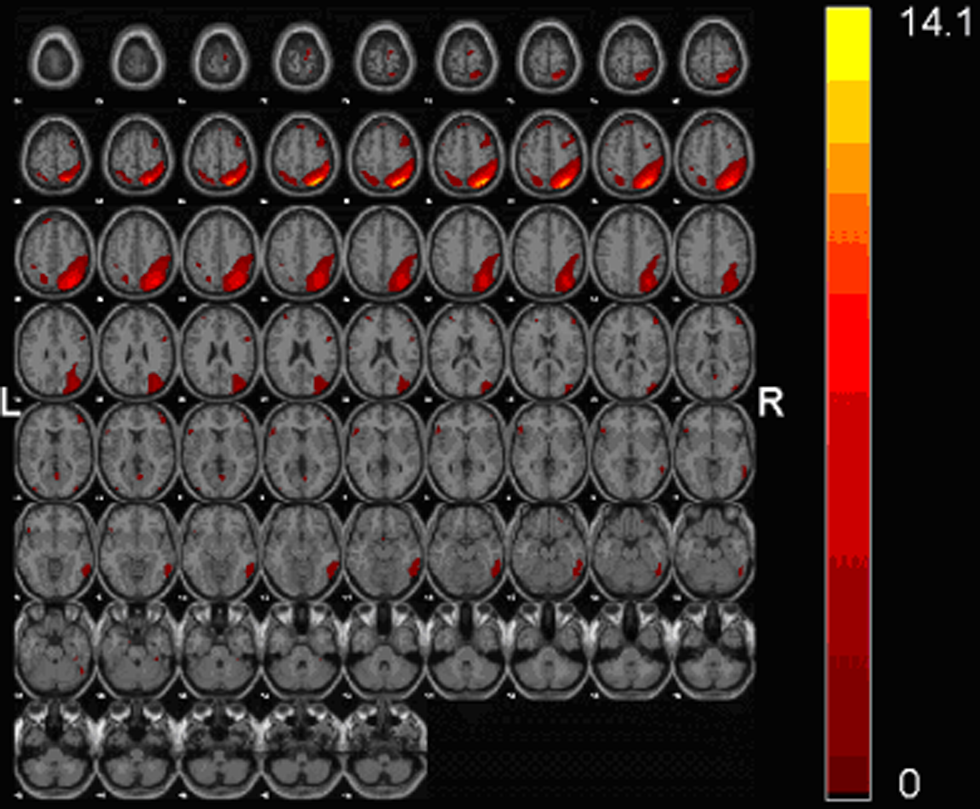

Supplement: Figure S39 — Spatial map of IC39. (TIF) [file pone.0025423.s039.tif]

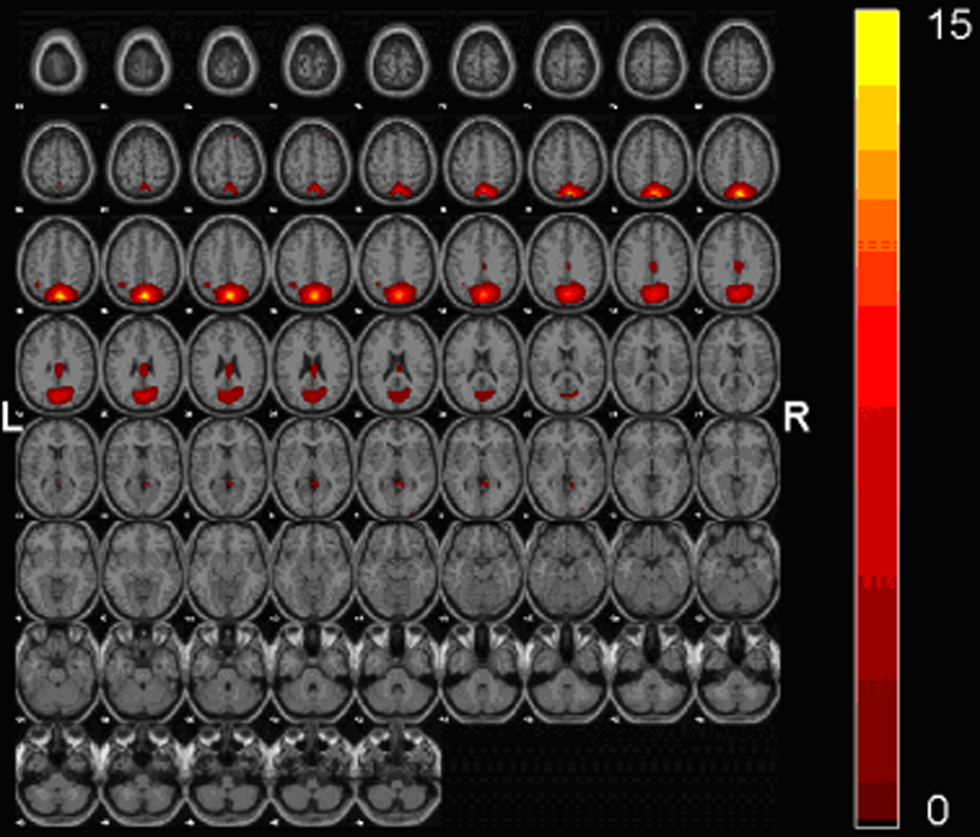

Supplement: Figure S40 — Spatial map of IC40. (TIF) [file pone.0025423.s040.tif]

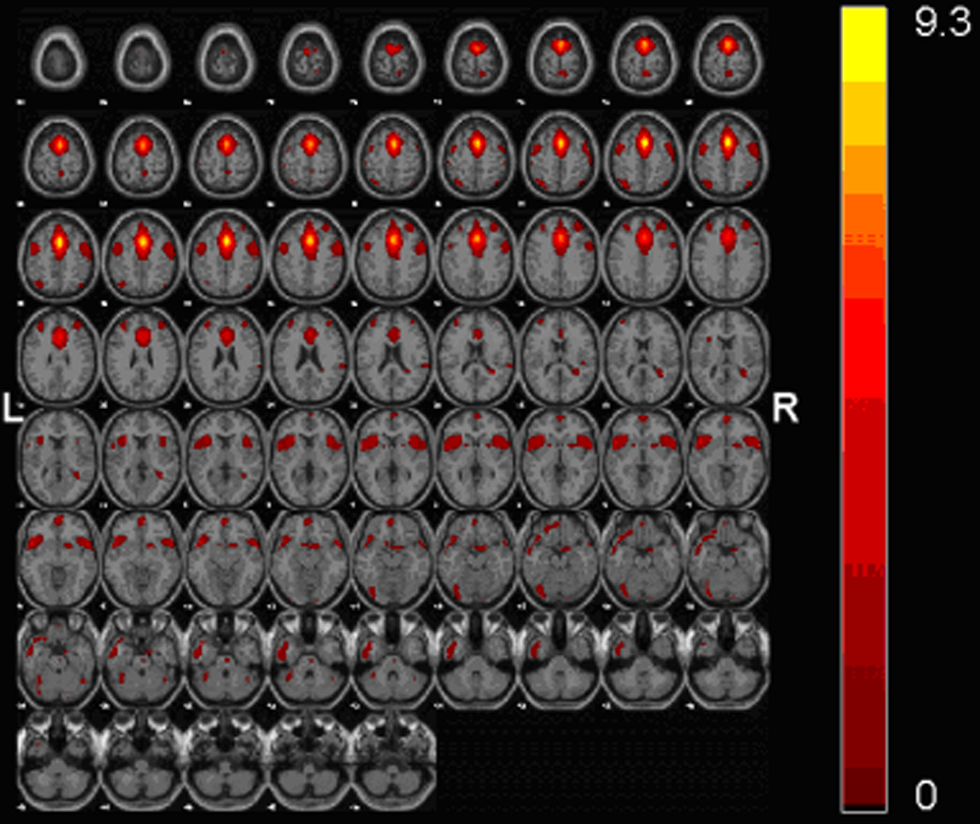

Supplement: Figure S41 — Spatial map of IC41. (TIF) [file pone.0025423.s041.tif]

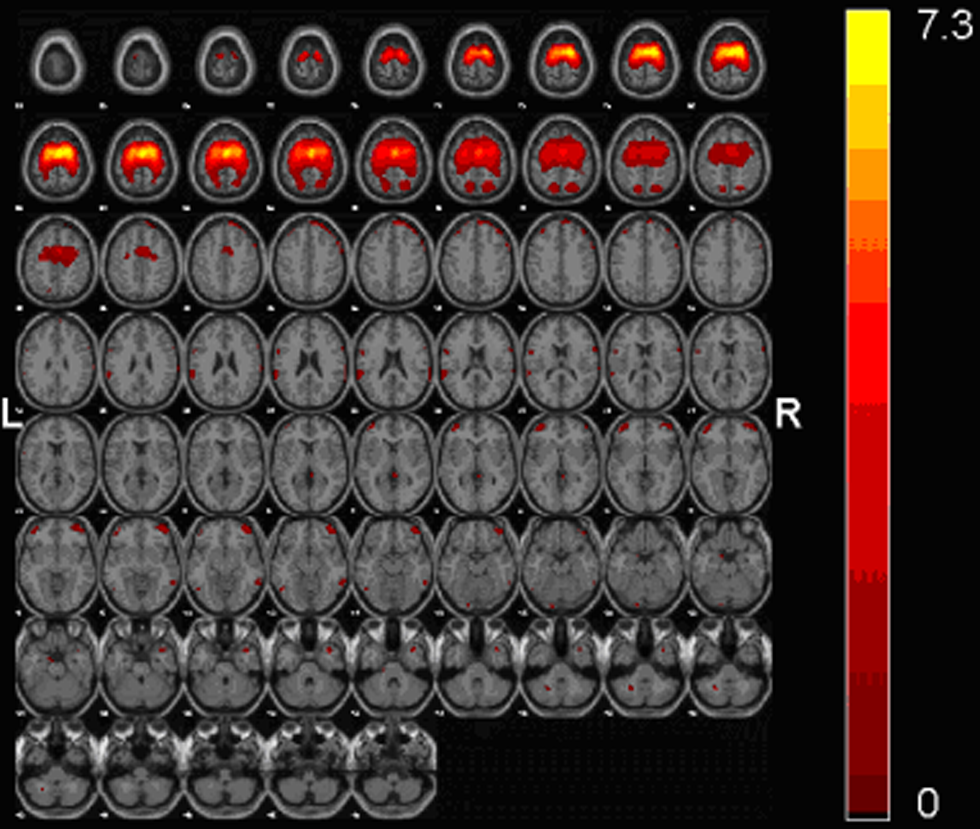

Supplement: Figure S42 — Spatial map of IC42. (TIF) [file pone.0025423.s042.tif]

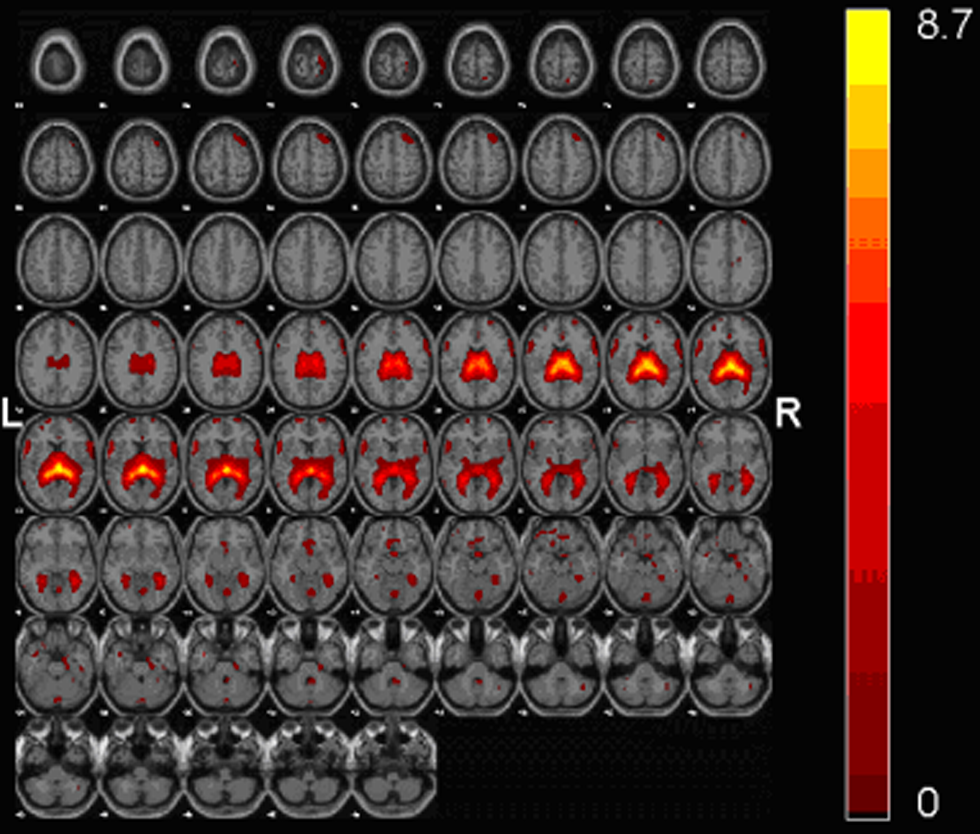

Supplement: Figure S43 — Spatial map of IC43. (TIF) [file pone.0025423.s043.tif]

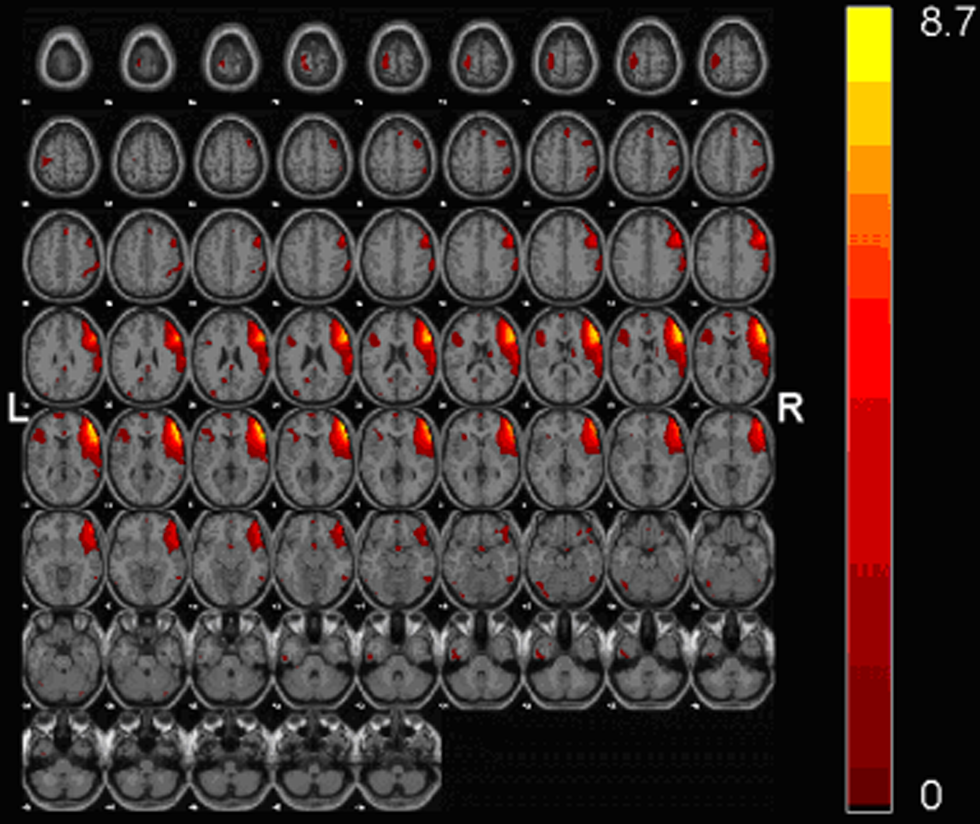

Supplement: Figure S44 — Spatial map of IC44. (TIF) [file pone.0025423.s044.tif]

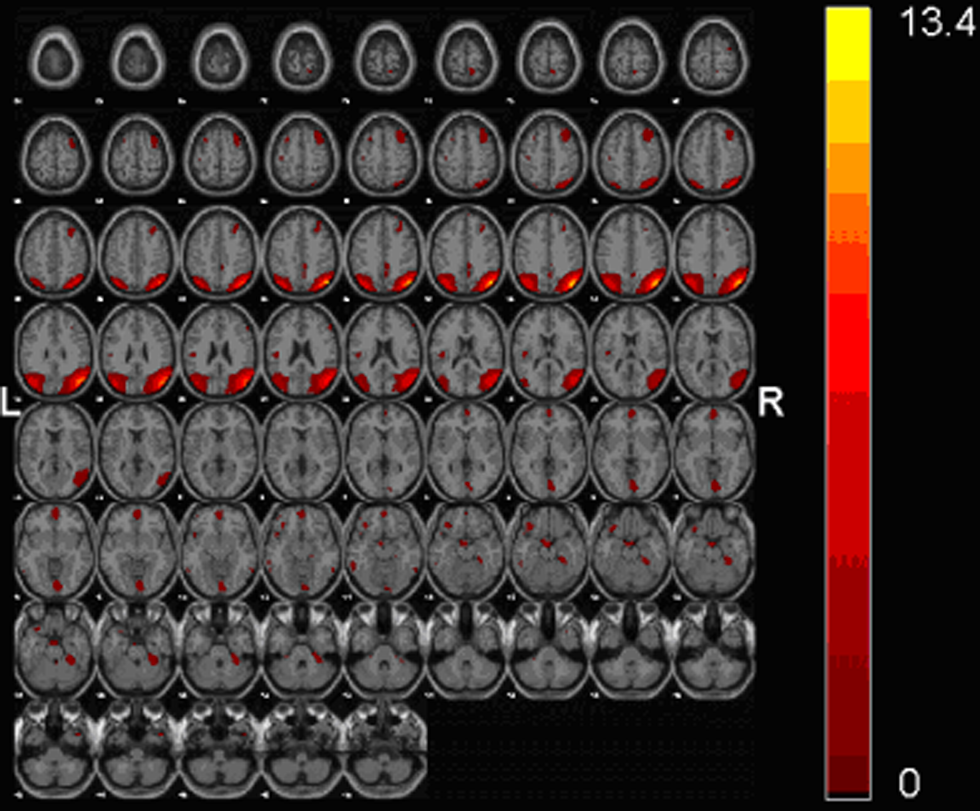

Supplement: Figure S45 — Spatial map of IC45. (TIF) [file pone.0025423.s045.tif]

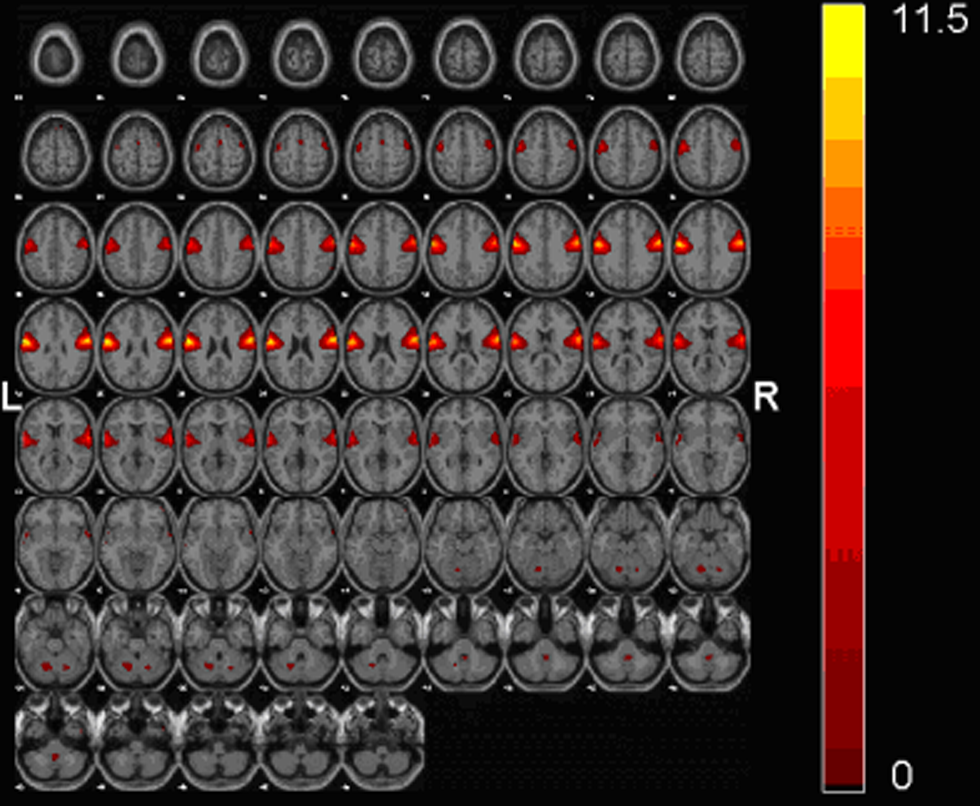

Supplement: Figure S46 — Spatial map of IC46. (TIF) [file pone.0025423.s046.tif]

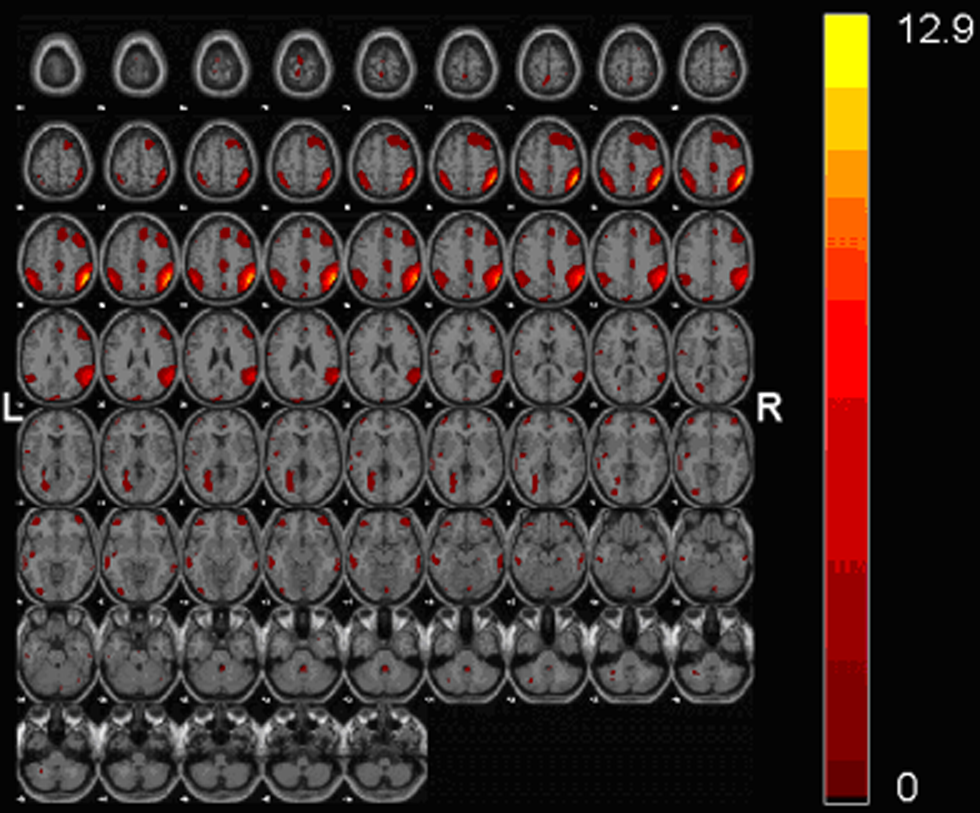

Supplement: Figure S47 — Spatial map of IC47. (TIF) [file pone.0025423.s047.tif]

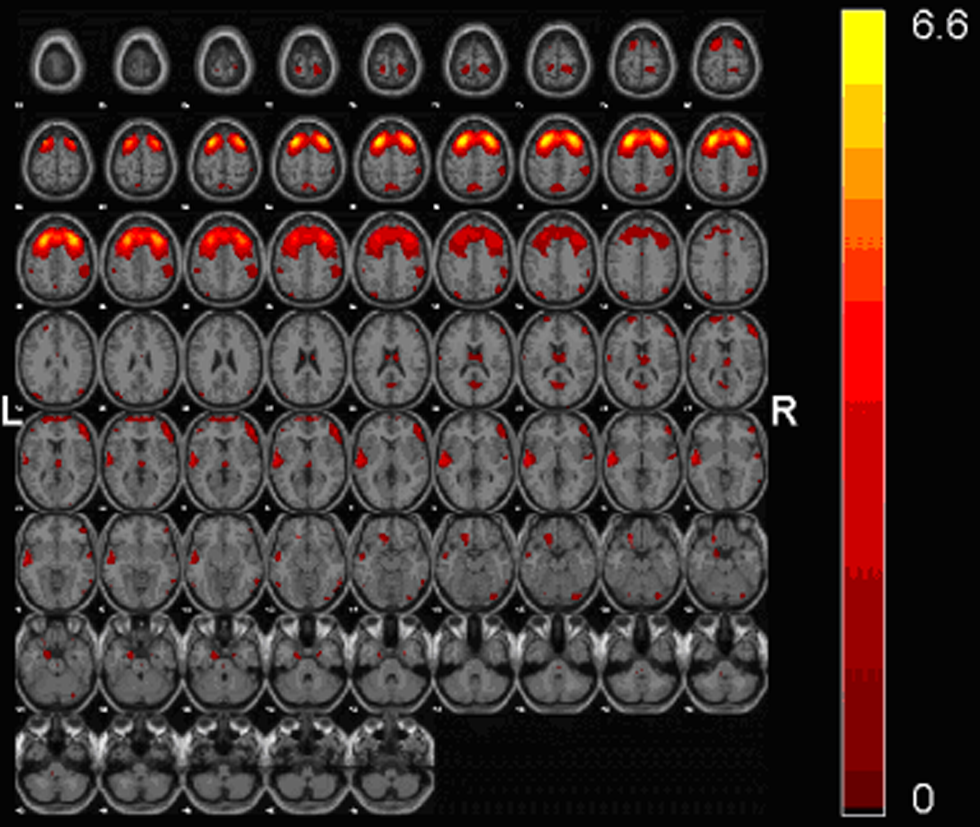

Supplement: Figure S48 — Spatial map of IC48. (TIF) [file pone.0025423.s048.tif]

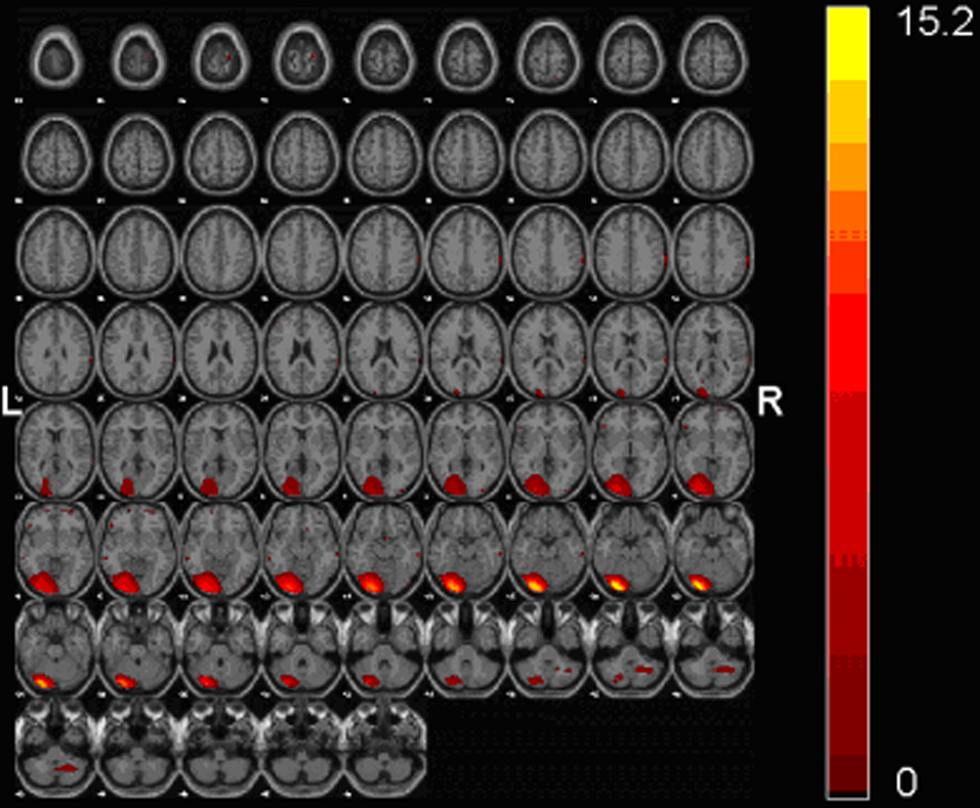

Supplement: Figure S49 — Spatial map of IC49. (TIF) [file pone.0025423.s049.tif]

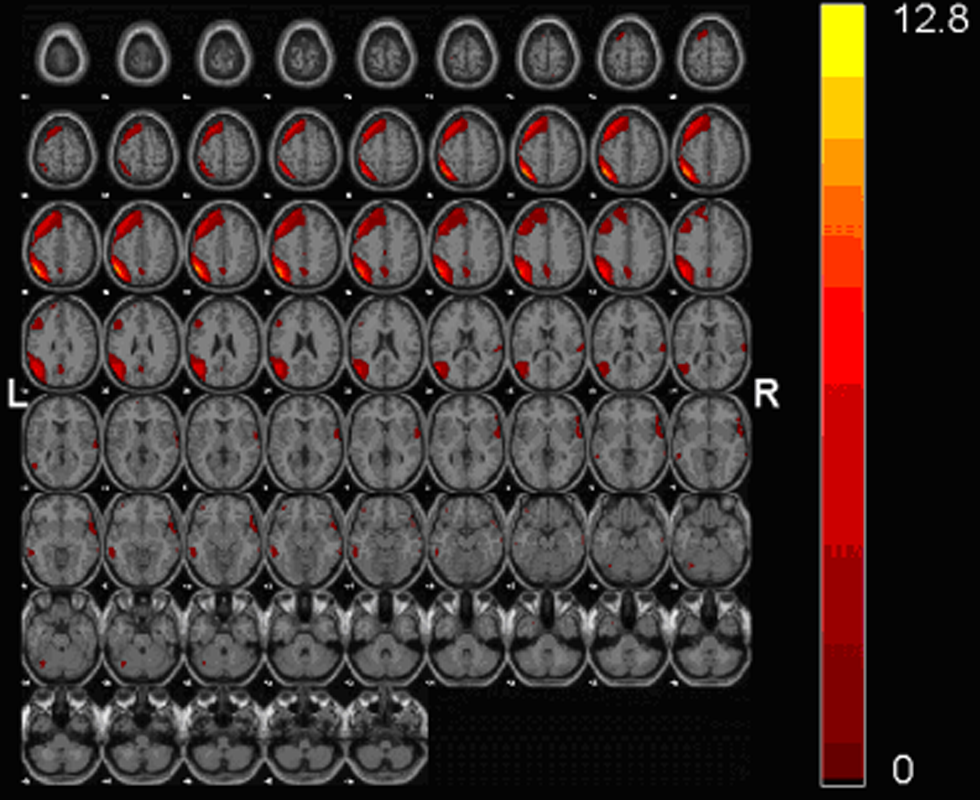

Supplement: Figure S50 — Spatial map of IC50. (TIF) [file pone.0025423.s050.tif]

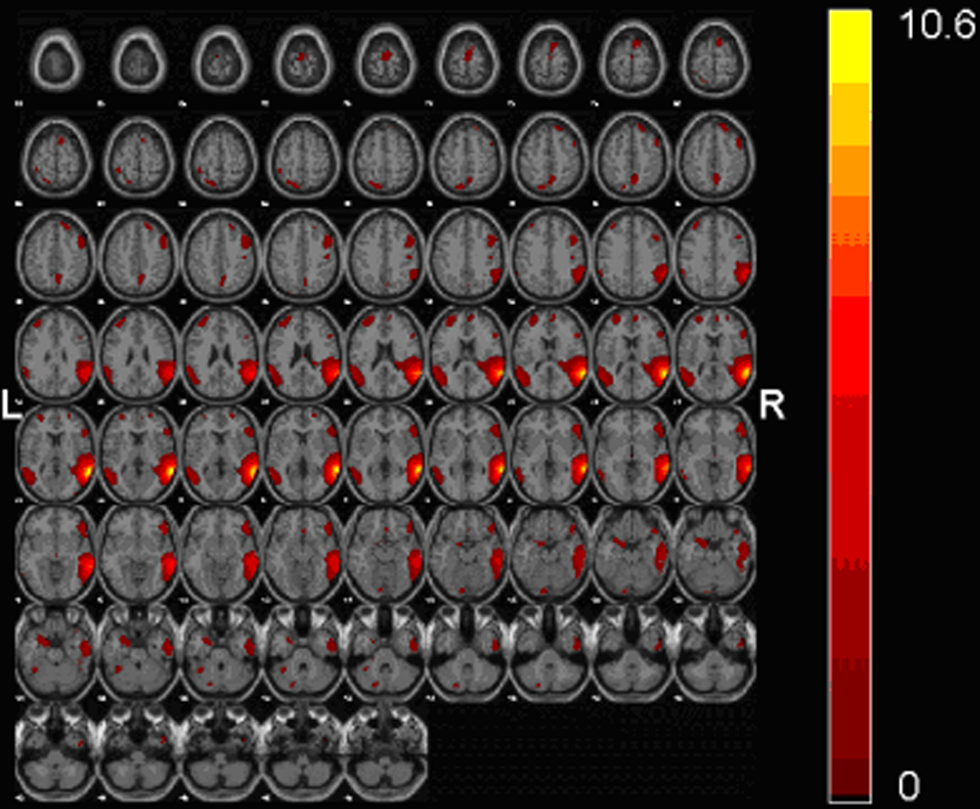

Supplement: Figure S51 — Spatial map of IC51. (TIF) [file pone.0025423.s051.tif]

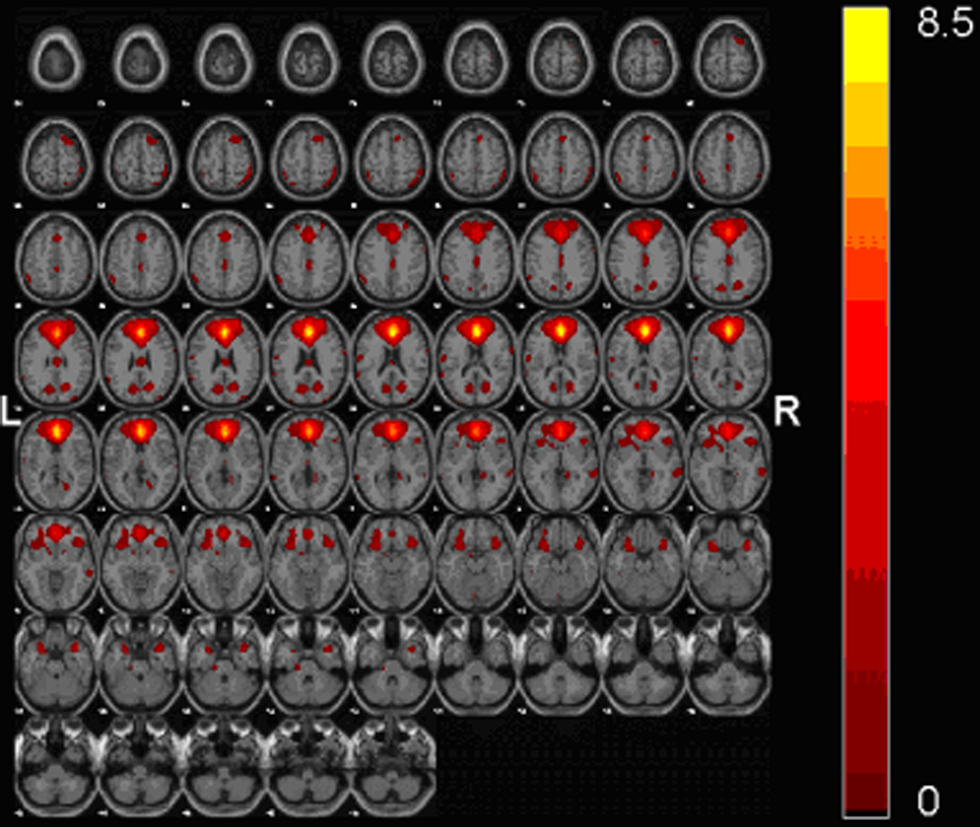

Supplement: Figure S52 — Spatial map of IC52. (TIF) [file pone.0025423.s052.tif]

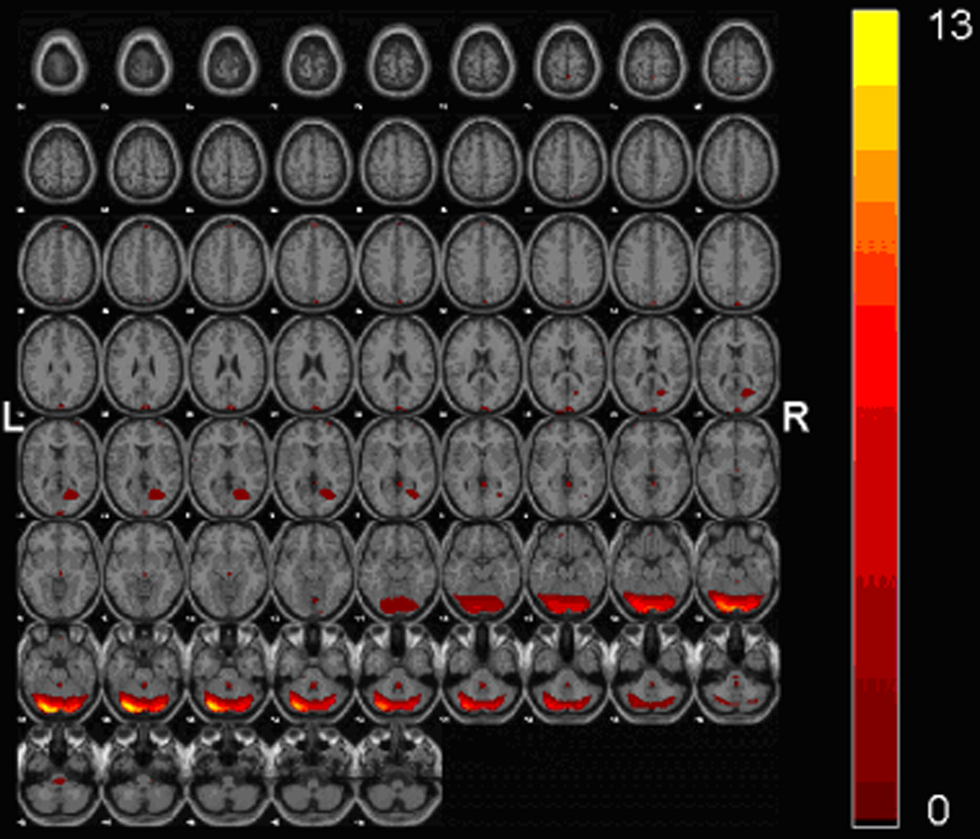

Supplement: Figure S53 — Spatial map of IC53. (TIF) [file pone.0025423.s053.tif]

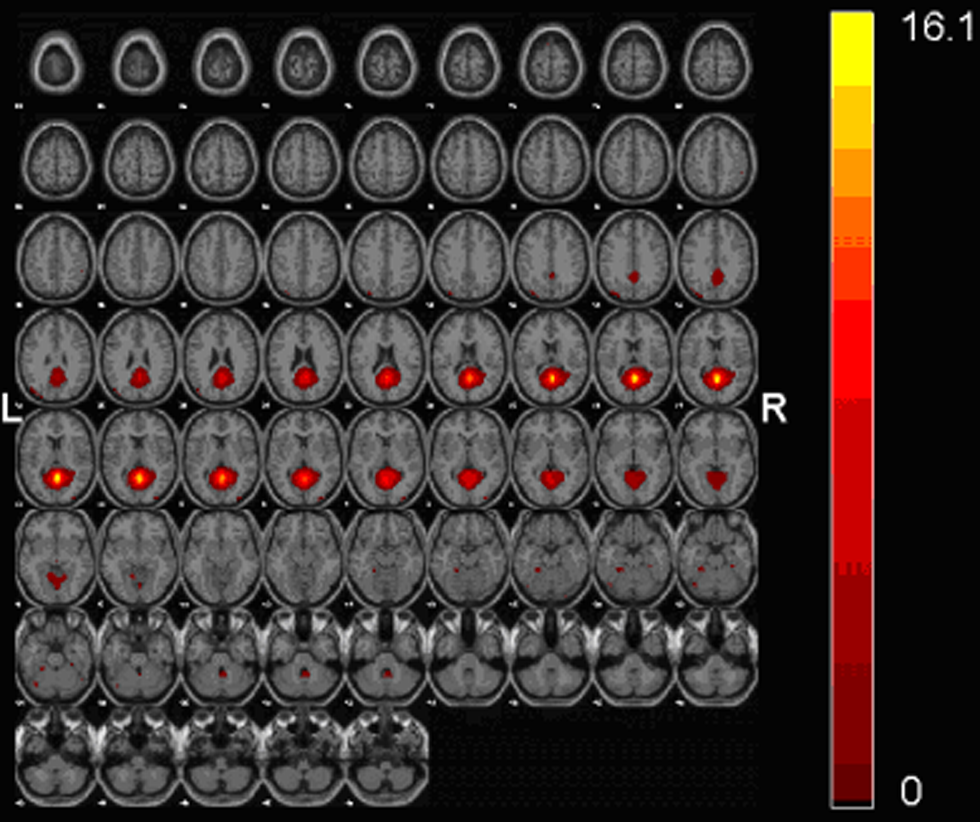

Supplement: Figure S54 — Spatial map of IC54. (TIF) [file pone.0025423.s054.tif]

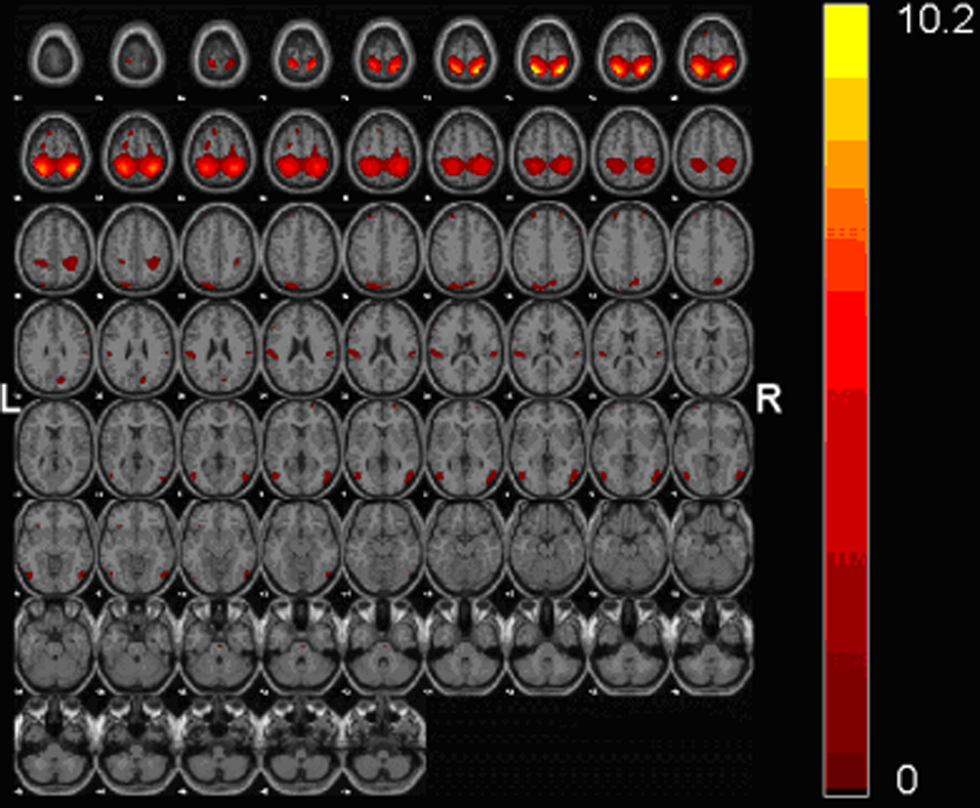

Supplement: Figure S55 — Spatial map of IC55. (TIF) [file pone.0025423.s055.tif]

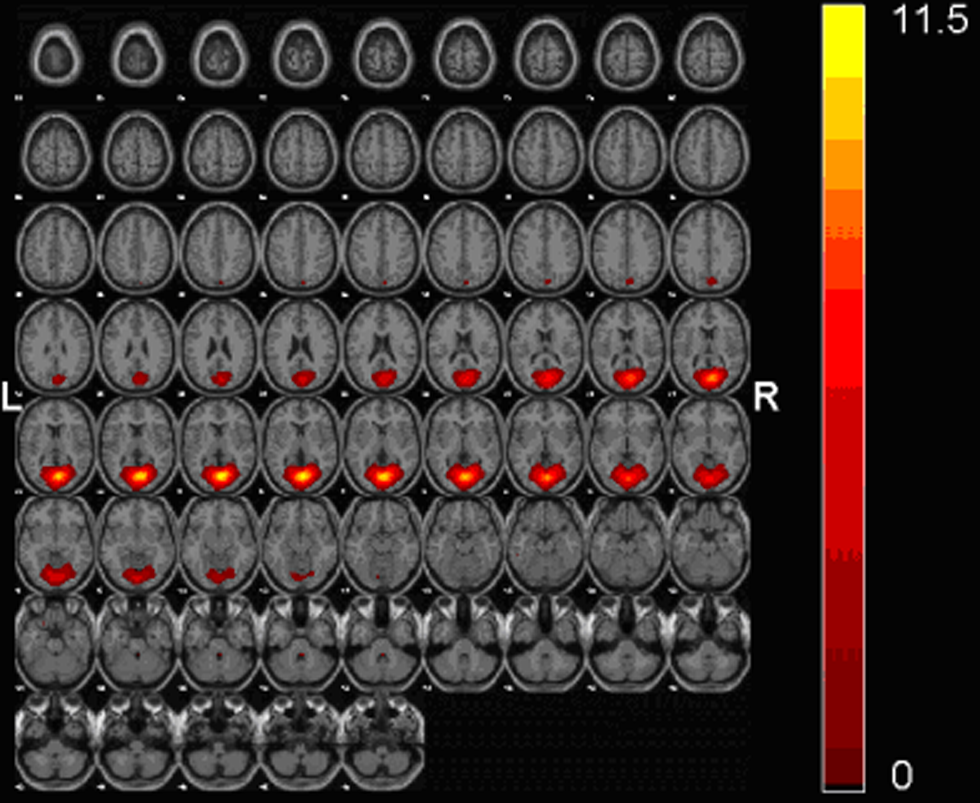

Supplement: Figure S56 — Spatial map of IC56. (TIF) [file pone.0025423.s056.tif]

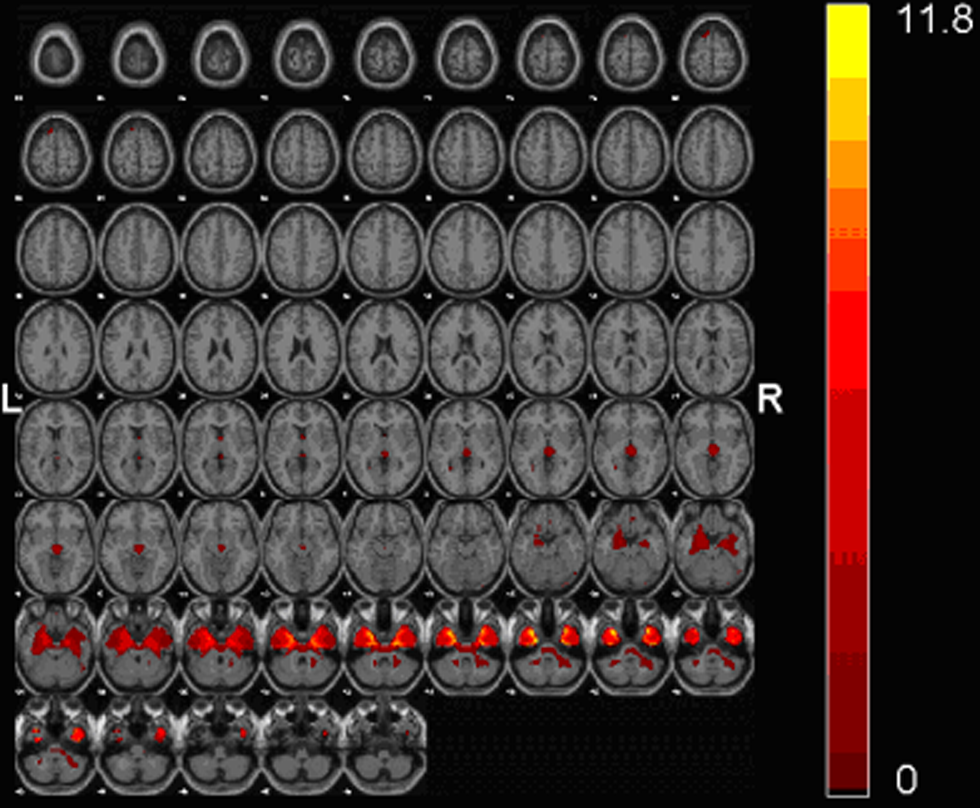

Supplement: Figure S57 — Spatial map of IC57. (TIF) [file pone.0025423.s057.tif]
